# Phosphorus-specific, liquid chromatography inductively coupled plasma mass spectrometry for analysis of inositol phosphate and inositol pyrophosphate metabolism

**DOI:** 10.1042/BCJ20253151

**Published:** 2025-11-04

**Authors:** Colleen Sprigg, Hayley L. Whitfield, Philip T. Leftwich, Hui-Fen Kuo, Tzyy-Jen Chiou, Adolfo Saiardi, Megan L. Shipton, Andrew M. Riley, Barry V.L. Potter, Dawn Scholey, Emily Burton, Mike R. Bedford, Charles A. Brearley

**Affiliations:** 1 School of Biological Sciences, University of East Anglia, Norwich, Norwich Research Park, NR4 7TJ, U.K; 2 Agricultural Biotechnology Research Center, Academia Sinica, Taipei, 115, Taiwan; 3 Laboratory for Molecular Cell Biology, University College London, London, WC1E 6BT, U.K; 4 Medicinal Chemistry & Drug Discovery, Department of Pharmacology, University of Oxford, Mansfield Road, Oxford, OX1 3QT, U.K; 5 School of Animal, Rural and Environmental Sciences, Nottingham Trent University, Southwell, NG25 0QF, U.K; 6 AB Vista, Marlborough, SN8 4AN, U.K

**Keywords:** animal nutrition, cell signalling, poultry physiology

## Abstract

Inositol phosphate (InsP) and diphosphoinositol phosphate (PP-InsP) analysis in tissues is plagued by multiple difficulties of sensitivity, regioisomer resolution and the need for radiolabelling with metabolic precursors. We describe a liquid chromatography (LC) inductively coupled plasma (ICP) mass spectrometry (MS) method (LC-ICP-MS) that addresses all such issues and use LC-ICP-MS to analyse InsPs in avian tissues. The highly sensitive technique tolerates complex matrices and, by powerful chromatography, resolves in a single run multiple non-enantiomeric *myo*-inositol tetrakisphosphates, *myo*-inositol pentakisphosphates and all inositol hexakisphosphates, including *myo*-inositol 1,2,3,4,5,6-hexakisphosphate (phytate), known in nature. It also separates and quantifies diphospho *myo*-inositol pentakisphosphate (PP-InsP_5_) isomers from their biological precursors and from 1,5-bis-diphospho myo-inositol 2,3,4,6 tetrakisphosphate (1,5-[PP]_2_- InsP_4_). Gut tissue InsPs, belonging to a non-canonical, lipid-independent pathway, are shown to differ from phytate digestion products and to be responsive to diet.

## Introduction

Inositol phosphates (InsPs) are canonical agents of intracellular signalling, with diverse cell biological function [1–3]. Of the 63 possible isomers of phosphate monoester-substituted *myo*-inositol, discrete function is assigned to a handful of species. Of these, inositol 1,2,3,4,5,6-hexakisphosphate, InsP_6_, phytate, is the most abundant InsP in the biosphere. Phosphorylation of its monoester substituents gives rise to diphosphoinositol phosphates (PP-InsPs), the biological functions of which are reviewed [4]. Consequently, InsP_6_ has a central position in the flux of inositol between inositol pentakisphosphate (InsP_5_) and PP-InsP pools. It is important therefore that analysis of InsP metabolism shows the relationship between InsP_5_, InsP_6_ and PP-InsP pools. Here, our understanding of the roles of InsP_6_, PP-InsPs and other InsPs rests heavily on analysis of cell lines. The behaviour of these lacks tissue context.

Among InsPs, InsP_6_ is a potent antinutrient of animals and human populations on subsistence diets and a significant risk factor in iron deficiency anaemia [5,6]. The enzymes that interconvert InsPs, PP-InsPs and their inositol precursor have been found to participate in inflammatory responses [7–9] and pathologies including cancer [10], diabetes [11], chronic kidney disease [12,13], colitis [14] and reproductive disorders [15], reviewed (1, 2, 4). Even so, despite numerous studies implicating InsPs and PP-InsPs in disease, there have been remarkably few descriptions of the InsPs and PP-InsPs of native tissues and organs and their response to therapeutic agents or environment including diet and metabolic insult.

The dearth of tissue analyses is in part due to the technical difficulty of measuring multiple stereoisomers at low concentrations. Radiolabelling of primary cells or tissue slices is an alternative that has also been applied in cell lines, unicellular organisms, including algae, yeast and protists and multicellular organisms, predominantly plants. The use of metabolic tracers, *myo*-[^3^H]-inositol or [^32^P]-orthophosphate, bears the caveat of assumption of labelling to equilibrium. Alternatively, non-equilibrium labelling studies have defined pathways of synthesis of InsPs. Even so, radiolabelling is time-consuming and comes with regulatory constraints.

The opportunity to assign identity to, and to measure, InsPs and PP-InsPs without labelling has always been recognized as an imperative – even if not readily attainable. The metal-dye-detection (m-d-d) HPLC method of Mayr [16] offers sensitivity at low pmol levels but sample work-up is involved and time-consuming. Capillary electrophoresis mass spectrometry (CE-MS) offers fmol sensitivity at the cost of both time and concentration/pre-purification of InsPs and PP-InsPs on TiO_2_ [17]. A corollary is that CE-MS is unsuitable for crude or complicated matrices. CE-MS further demands challenging organic syntheses of ^13^C standards (isotopologues) for calibration and for confirmation of identity of peaks and has yet to be successfully adopted beyond the originating laboratory. Consequently, with the explosion of interest in PP-InsPs (1, 2, 4), which constitute a tiny mole fraction of the total InsP (and PP-InsP) content of tissues, there is a need for alternative approaches that can vouchsafe the intricacies of InsP and PP-InsP function without the constraints of radiolabelling or pre-purification.

An ability to handle complicated or crude matrices is desirable because these are information rich. InsPs and PP-InsPs are commonly extracted from animal cells and tissues with perchloric acid or trichloroacetic acid; from seeds, beans and grains with hydrochloric acid; from animal gut contents or faecal matter with NaF-EDTA; and from soil matrices with NaOH-EDTA. From a chromatographic perspective, these extractants are generally considered not compatible with LC-MS or CE-MS, either because of the constraints of column chemistry or the extreme sensitivity of CE to ionic content. From a detection perspective, electrospray MS detection is susceptible to ion suppression effects. Consequently, both conventional LC-MS and CE-MS demand exchange of extractant for more benign loading solutions. The much higher sample loading available on anion-exchange LC (hundreds of microlitres) compared with CE (a few nanolitres) is a potential advantage of the former method.

Herein, we elaborate on how liquid chromatography inductively coupled plasma mass spectrometry (LC-ICP-MS) allows measurement of InsPs and PP-InsPs in crude or purified biological matrices across taxa. The interpretation of chromatography is remarkably simple, as is the nature of detection: detector response is proportional to phosphorus content, and the detector signal does not require complicated de-convolution.

## Results

### Chromatographic resolution of multiple InsP_4_, InsP_5_, InsP_6_ and PP-InsP isomers on a single gradient

**Figure 1:**
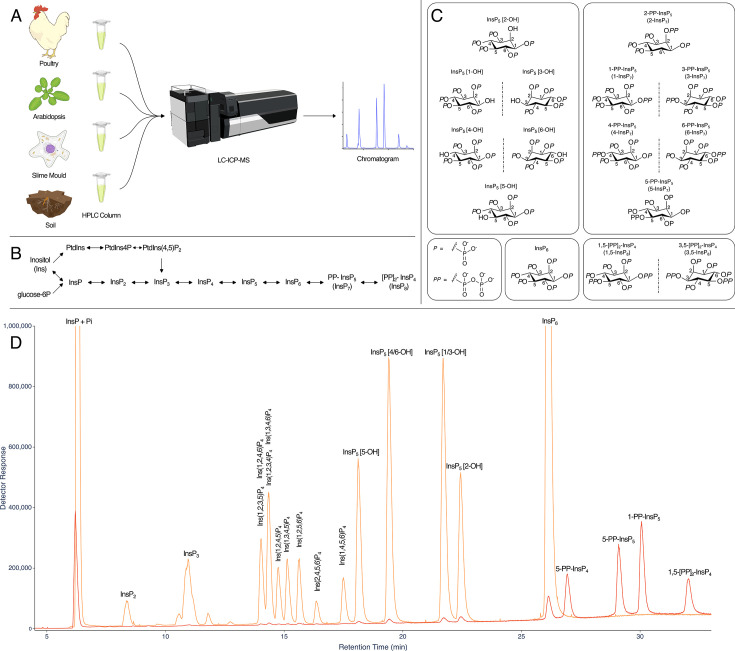
Separation of PP-InsPs and InsPs by LC-ICP-MS. **A**. Cartoon of an LC-ICP-MS procedure applicable to diverse taxa and sample matrices. **B**. Simplified metabolic relationships of InsPs, PtdInsPs and PP-InsPs showing lipid-derived and ‘soluble’ contributions to PP-InsP synthesis. Arrows indicate that pools of metabolites at individual levels of phosphorylation are in exchange with each other. For some pools, the interconverting enzymes are reversible whereas for others different enzymes may operate. **C**. The complexity of InsP_5_ and PP-InsP_5_ speciation. For each, there are six possible stereoisomers of which four exist as two pairs of enantiomers (reflected here in a mirror plane). For both, the other two isomers (like InsP_6_) are *meso*-compounds: they possess an internal plane of symmetry between carbon 2 and carbon 5. **D**. Analysis of an acid hydrolysate of *myo*-InsP_6_ (orange), and a 50-fold dilution thereof ‘spiked’ with 5-PP-Ins(1,3,4,6)P_4_ (5-PP-InsP_4_), 5-PP-InsP_5_, 1-PP-InsP_5_ and 1,5-(PP)_2_-InsP_4_ (red line). Samples were run on a CarboPac PA200 HPLC column eluted with a shallow linear gradient of HCl. Equivalent separations of the inositol pyrophosphate species from InsP_4_, InsP_5_ and InsP_6_ species shown here have been observed on more than 30 occasions on CarboPac PA200 column coupled to ICP-MS.

**Figure 2:**
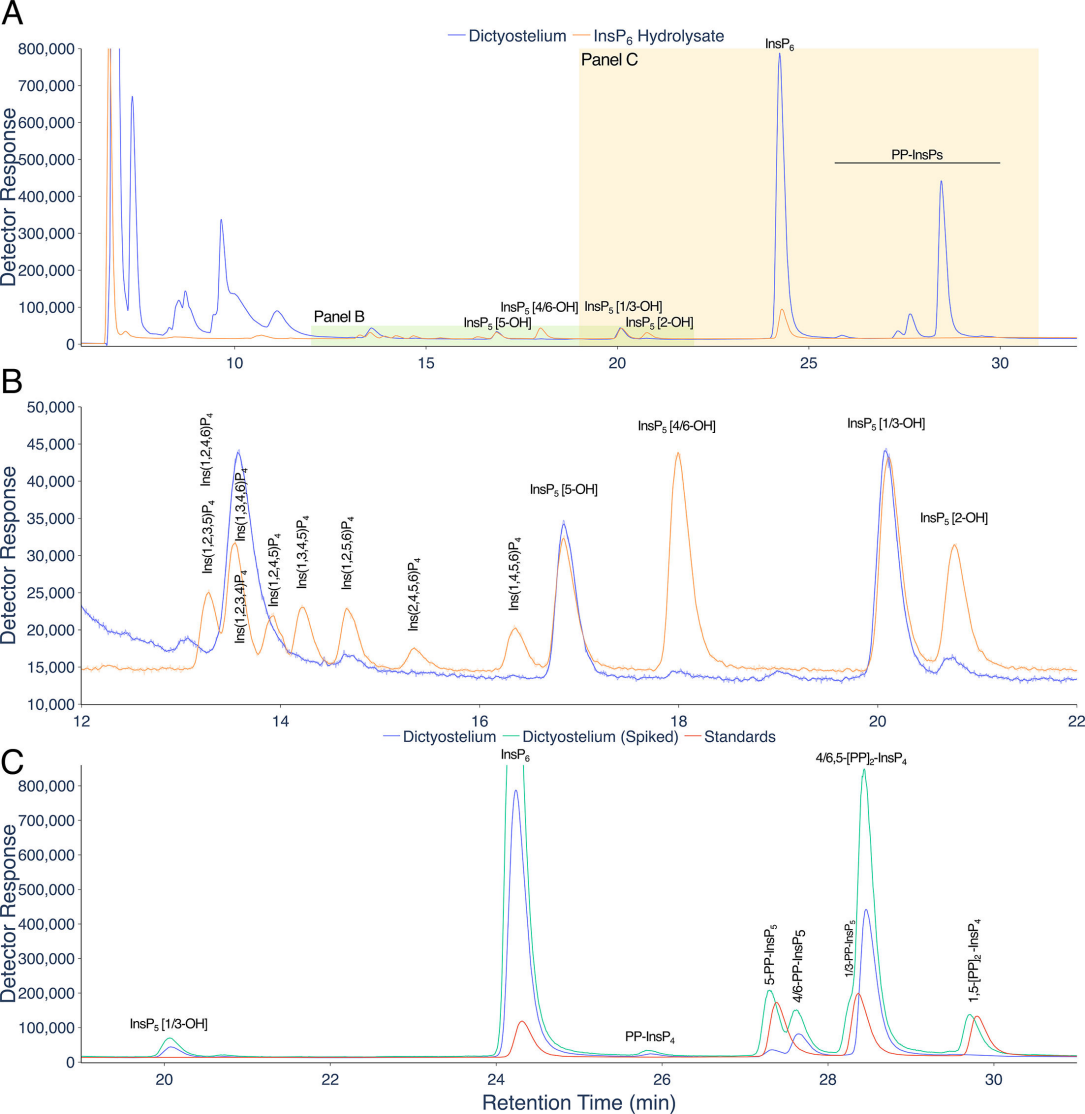
Separation of PP-InsPs and InsPs in *Dictyostelium* by LC-ICP-MS. **A**. A perchloric acid extract of *Dictyostelium discoideum* amoebae (blue trace) and an acid-hydrolysate of InsP_6_ (orange trace). **B**. an expansion of the InsP_4_ and InsP_5_ region of the chromatogram shown in A. **C**. An expansion of the InsP_6_ and PP-InsP region of the chromatogram shown in A (*Dictyostelium* extract, blue trace), with the same sample (at different concentration) spiked with 1-PP-InsP_5_, 5-PP-InsP_5_ and 1,5-(PP)_2_-InsP_4_ (green trace), and separately a set of the standards of InsP_6_, 1-PP-InsP_5_, 5-PP-InsP_5_, 1,5-(PP)_2_-InsP_4_ (red trace). Compounds were resolved on a CarboPac PA200 column eluted with a positive exponential gradient of HCl. The PP-InsP peaks for which standards were not available, 4/6-PP-InsP_5_ and 4/6,5-(PP)_2_-InsP_4_, are identified according to the known order of elution of PP-InsP_5_s and 1/3-PP-InsP_5_ eluted on CarboPac PA200 ^22,23^ and by reference to the characterization of *Dictyostelium* by CE-MS ^17^ which identifies 4/6,5-(PP)_2_-InsP_4_ as the principal PP-InsP species therein. Chromatography with resolution similar to this was observed on more than five different injections of different *Dictyostelium* samples.

ICP-MS is a powerful approach for elemental analysis but is rarely coupled to liquid chromatography [18]. In contrast, MS and tandem MS-MS are commonly coupled to liquid chromatography in pharmaceutical and biomedical contexts, while CE-MS use is much less common across disciplines. The example taxa/matrices analysed in this manuscript are shown (Figure 1A). Each demands different extraction regimes. The different InsPs and PP-InsPs contained therein can be analysed by a single chromatographic approach (LC) coupled to phosphorus-specific detection (ICP-MS) and can all be placed within generic pathways of higher InsP and PP-InsP synthesis, whether derived from lipid or ‘soluble’ precursors (Figure 1B). The structures and enantiomeric relationships of all *myo*-InsP_5_, InsP_6_ and PP-InsP_5_ isomers are shown (Figure 1C). Separation of InsPs and PP-InsPs bearing between two and eight phosphates is possible in a single chromatographic run with detection of the phosphorus content by ICP-MS (Figure 1D). Like CE-MS [17], LC-ICP-MS identifies 1/3-PP-InsP_5_, 4/6-PP-InsP_5_, 5-PP-InsP_5_, 1,5-(PP)_2_-InsP_4_ and 4/6,5-(PP)_2_-InsP_4_ in *Dictyostelium discoideum* (Figure 2) but simultaneously measures multiple InsP_4_ species and all InsP_5_ species. We note the resolution of a minor peak eluting shortly after InsP_6_ at approximately 26 min that is most likely an endogenous PP-InsP_4_, of unknown regiochemistry. Without chiral stationary phases or chiral shift reagents, neither CE-MS nor LC-ICP-MS resolve enantiomers, viz. 1-PP-InsP_5_ and 3-PP-InsP_5_ or 4-PP-InsP_5_ and 6-PP-InsP_5_ named with 1/3- and 4/6- prefixes in the preceding sentence. Similarly, it is not possible to resolve Ins(2,3,4,5,6)P_5_, hereafter InsP_5_ [1-OH], from Ins(1,2,4,5,6)P_5_, hereafter InsP_5_ [3-OH], or Ins(1,2,3,5,6)P_5_, hereafter InsP_5_ [4-OH], from Ins(1,2,3,4,5)P_5_, hereafter InsP_5_ [6-OH]. We use the term InsP_5_ [1/3-OH] and InsP_5_ [4/6-OH] where the speciation of enantiomers is unknown.

**Figure 3:**
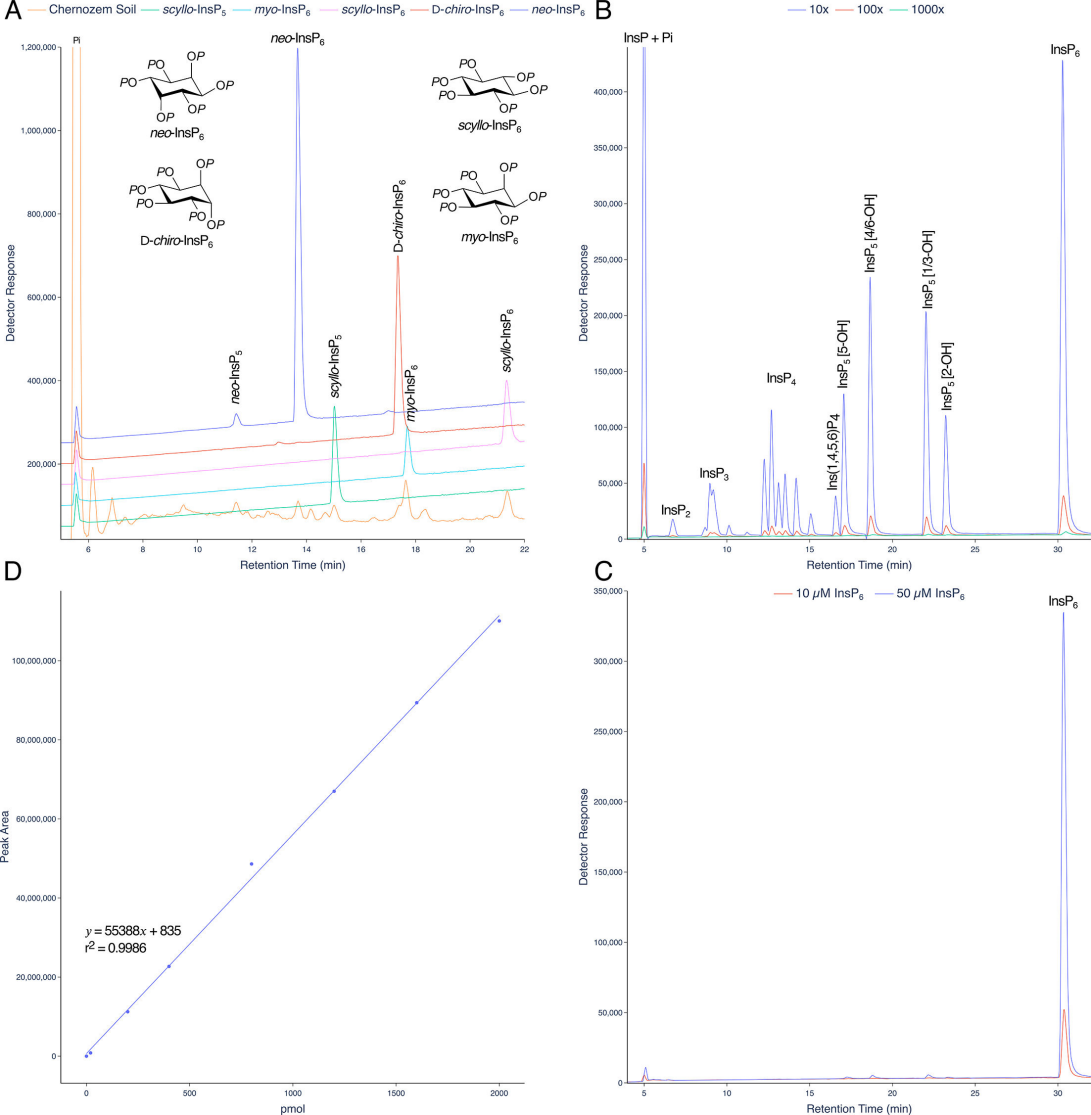
Resolution of inositol hexakisphosphates in soil. **A**. InsPs from a chernozem soil analysed on a short, steep linear gradient (orange line). Individual standards of *neo*-InsP_6_ (blue), D-*chiro*-InsP_6_ (red), *myo*-InsP_6_ (cyan) and *scyllo*-InsP_6_ (green) are shown with their chemical structures. Resolution of soil samples equivalent to this has been observed on more than 100 occasions by LC-ICP-MS. **B**. A three-decade dilution of an InsP_6_ hydrolysate. The aliquots injected are 10-, 100- and 1000-fold dilutions of the hydrolysate analysed in Figure 2C (analysed as 20 μl injection). The peak areas (counts.min) of the InsP_6_ peak are 8,249,418, 813,719 and 86,360, respectively. **C**. Injections (10 μl) of InsP_6_ dodecasodium salt. The peak areas (counts.min) of the InsP_6_ peak are 6,301,172 at 50 μM and 1,055,441 at 10 μM. **D**. A calibration curve for phosphorus (detector response = peak area). Different amounts of phosphorus (single samples) were injected, as NaH_2_PO_4_, and the Pi peak integrated. For B, C and D, single samples were analysed. Reproducibility for biological samples is further described in Supplementary Figure S5.

Among biological matrices, soils are unique in the breadth of inositol hexakisphosphate species present. A direct comparison of LC-ICP-MS and ^31^P NMR for analysis of InsPs in a Swedish podsoil was made [19], with detector response for phosphorus differing by less than 7% across the HPLC gradient. Here we show how a short, steeper linear gradient of HCl also resolves all known naturally occurring inositol hexakisphosphates, *neo*-InsP_6_, D-*chiro*-InsP_6_, *myo*-InsP_6_ and *scyllo*-InsP_6_, present in a Chernozem soil sample, in addition to both *neo*-InsP_5_ and *scyllo*-InsP_5_ (Figure 3). Chromatograms for dilutions of an InsP_6_ hydrolysate or InsP_6_ standard are shown (Figure 3B C). A calibration curve for detection of phosphorus (phosphate) is shown (Figure 3D).

### LC-ICP-MS identifies InsPs in complicated matrices without pre-purification of analytes

**Figure 4:**
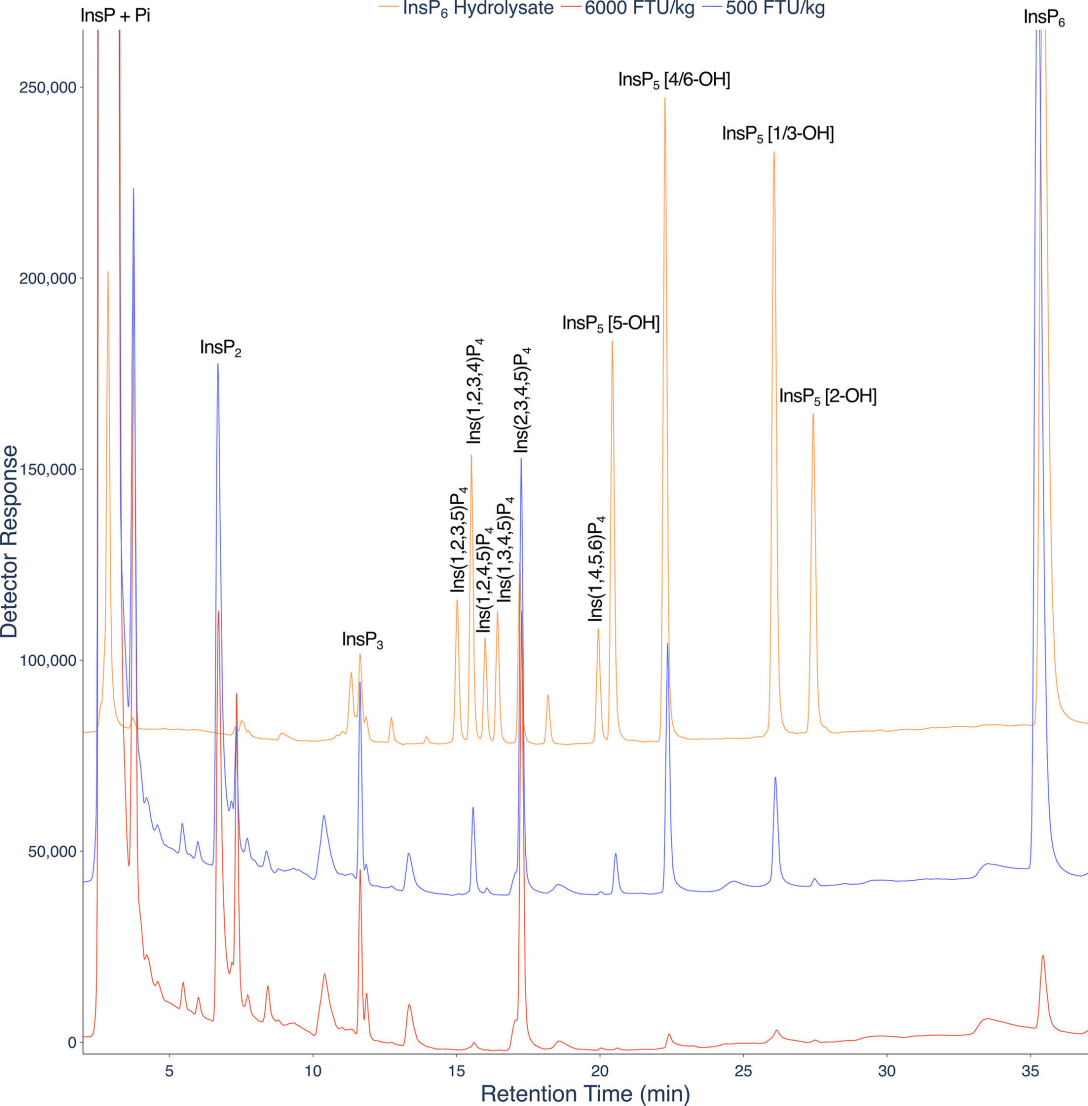
**LC-ICP-MS analysis of InsP_6_ digestion in the avian gastrointestinal tract**. LC-ICP-MS of lumenal ileal content of birds fed a diet containing low (500 FTU/kg) (blue) or high (6000 FTU/kg) (red) phytase. A hydrolysate of InsP_6_ is shown (orange). The extract was resolved on a CarboPac PA200 column eluted with methanesulfonic acid. These individual chromatograms are representative, in terms of retention time and signal to noise ratio, of seven different tissue samples analysed in the middle of a set of more than 50 consecutive injections.

CE-MS of InsPs in mammalian tissues, cell lines, mouse tissues, plants, amoebae and yeast is dependent on extraction with HClO_4_ and pre-purification on TiO_2_ [17]. Analysis of crude extracts offers complementary information, as in description of dietary influence on InsPs of the gut lumen of chicken where simple NaF-EDTA extraction at alkaline pH is common [20,21]. Here, we treat the gut lumen as an organ, a concept widely accepted in modern microbiome-health contexts [22]. Accordingly, we first analysed InsPs in NaF-EDTA extracts of gastro-intestinal contents (digesta) of gizzard and ileum of birds fed two levels of phytase in their diet. InsP_2_ (isomers unidentified), InsP_3_ (isomers unidentified), Ins(1,2,3,4)P_4_, Ins(1,2,4,5)P_4_, Ins(2,3,4,5)P_4_, Ins(1,4,5,6)P_4_, InsP_5_ [5-OH], InsP_5_ [4/6-OH], InsP_5_ [1/3-OH] and InsP_6_ were detected in digesta of birds fed the lower (500 FTU/kg) dose of phytase by LC-ICP-MS (Figure 4, Supplementary Figure S1A,B). At 6000 FTU/kg, InsPs were barely detectable, having been fully degraded.

For comparison with established methodology, gizzard lumenal samples were also analysed by HPLC-UV (Supplementary Figure S1C). This method reports on analytes that interact with ferric ion. While these include InsPs and PP-InsPs [23,24], other analytes that chelate or engage in Fenton chemistry with ferric ion in the acid conditions of the chromatography will interfere. These potentially include catechols, diarylcyclopentenones and flavonoids. As these molecules lack phosphorus, they do not interfere with LC-ICP-MS. For comparison, the LC-ICP-MS data (Supplementary Figure S1A,B) were obtained from 10 μl of a 10-fold diluted NaF-EDTA extract while the HPLC-UV data (Supplementary Figure S1C) were obtained from 20 μl of the undiluted NaF-EDTA extract. LC-ICP-MS is approximately two orders of magnitude more sensitive. Ins(2,3,4,5)P_4_ is the predominant InsP in both the ileal and gizzard digesta analysed (Figure 4, Supplementary Figure S1).

The 6-phytase used in this feeding trial is a modified *E. coli*-derived enzyme. The first characterization of this enzyme was reported [25]. The principal pathway of dephosphorylation of phytate in the chicken digestive tract is shown (Supplementary Figure S1D) [20,21]. It is characterized by retention of the axial 2- phosphate at all levels of dephosphorylation as far as InsP. Because the birds were fed a mash diet, not heat-treated, additional endogenous phytase activity of the wheat-based diet (which possesses very high mature grain phytase activity [26]) is apparent in the generation of InsP_5_ [5-OH] in the gizzard lumen (Supplementary Figure S1A-C). InsP_5_ [5-OH] is a characteristic product of Triticeae (cereal, including wheat) purple acid phytase [27]. The effect of dietary treatment on lumenal InsP content of gizzard and ileum was published previously [28]. Digestion of dietary InsP_6_ liberates inositol, which passes by undefined mechanism to the blood. Inositol levels of jejunum tissue, kidney and blood correlate positively with lumenal inositol content of the jejunum [29]. Similarly, ileal lumen (digesta) inositol correlates positively with blood inositol [21]. These analyses highlight how digestion releases the inositol precursor of tissue InsP and PP-InsP synthesis into the blood.

### InsPs of duodenum, jejunum and ileum tissues can be analysed by LC-ICP-MS

**Figure 5:**
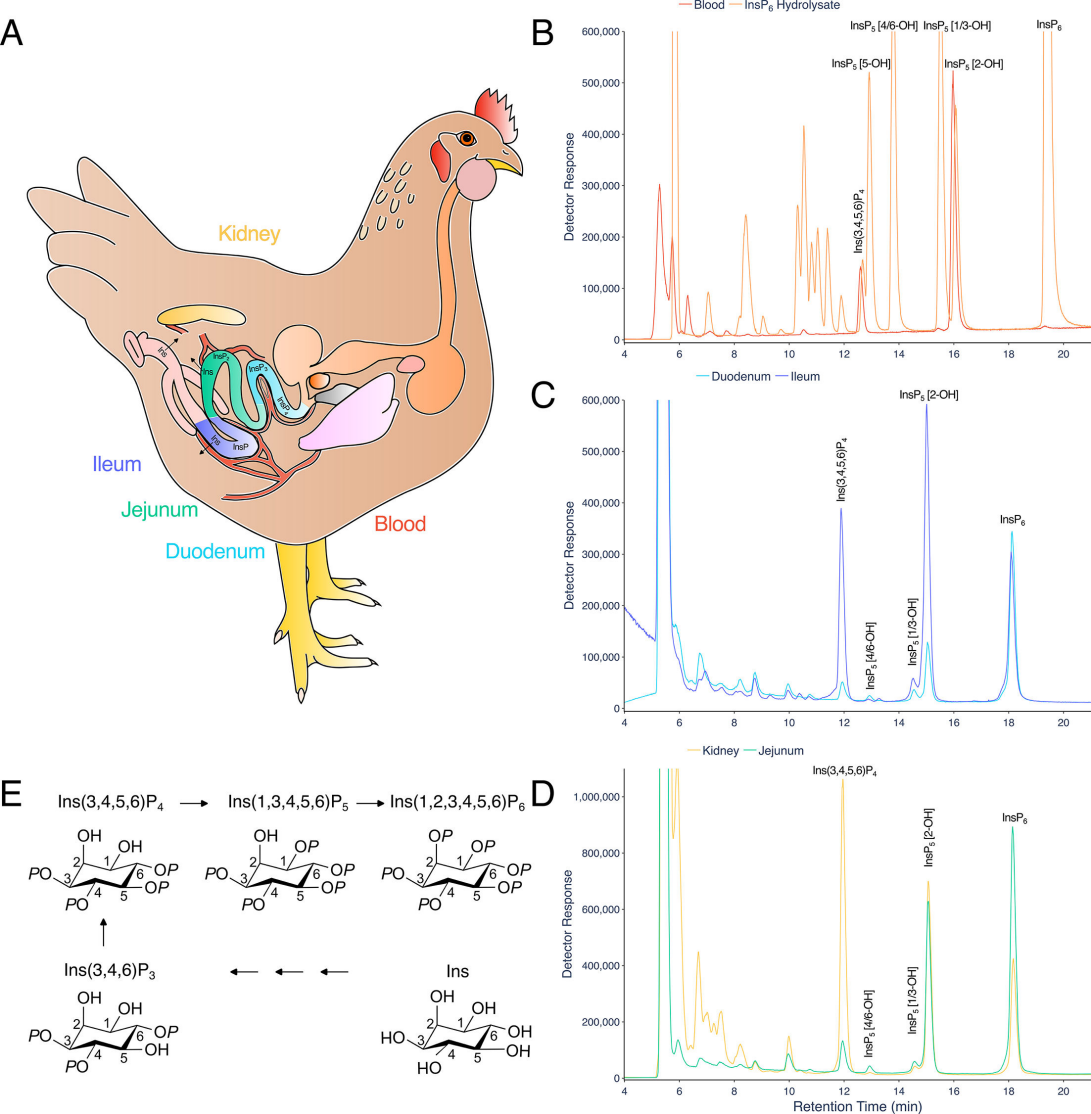
LC-ICP-MS analysis of tissue InsPs. **A**. Cartoon of chicken digestive tract and tissues analysed: kidney (yellow), duodenum (cyan), jejunum (green) and ileum (purple). **B**. InsPs extracted from blood are shown (red) and standards (InsP_6_ hydrolysate) are shown (orange). **C**. InsPs of tissues: duodenum (cyan), ileum (purple). **D**. InsPs of tissues: jejunum (green) and kidney (yellow). **E**. Established pathway of InsP_6_ synthesis in avian erythrocytes after Stephens and Downes ^30^. The identities of InsP_1_ and InsP_2_ species are not well characterized. For B, whole blood from a 35d-old broiler was extracted in perchloric acid and diluted with NaF-EDTA. For C and D, InsPs extracted from tissues with perchoric acid were concentrated on TiO_2_ prior to LC-ICP-MS. For B and C/D, chromatography was performed on separate HPLC machines on separate CarboPac PA200 columns eluted with gradients of HCl. The chromatograms shown were obtained from tissues of birds fed a control diet, lacking phytase. Separations of inositol phosphates on the CarboPac PA200 column matching the resolution shown have been observed on more than 100 occasions for blood, on more than 50 occasions each for duodenum, ileum, jejunum and kidney tissues by LC-UV. For the ICP-MS analysis shown, the coefficient of variation of retention time of e.g., the InsP_5_ [2-OH] peak is less than 1% across more than 10 runs across different tissues. A set of six blood samples is shown in Supplementary Figure S2.

Gut tissues are bounded on one side by the lumen and on the other by the bloodstream. The blood receives nutrients from the gut (Figure 5A). The hepatic portal vein drains blood from the gastrointestinal tract to the liver. The predominant cells of the blood are erythrocytes. Avian blood shares the same InsP species as the duodenum, jejunum and ileum (Figure 5B–D, Supplementary Figure S2) but lacks appreciable InsP_6_, which is a substantial component of duodenum, jejunum and ileum tissue InsPs. The blood InsP profile is consistent with m-d-d HPLC, NMR and radiolabelling [16,30,31], which revealed that the specific enantiomer Ins(3,4,6)P_3_ is the precursor of the specific enantiomer Ins(3,4,5,6)P_4_ and the latter is the precursor of Ins(1,3,4,5,6)P_5_, InsP_5_ [2-OH] [30]. We may assume that InsP_5_ 2-kinase catalyzes the final step of InsP_6_ synthesis as described for other animals, plants and yeast [32]. The liver, distal to the gut and the kidney share the same major species Ins(3,4,5,6)P_4_ and InsP_5_ [2-OH], again with lower levels of InsP_6_ (Figure 5D and [28]). InsP_5_ [2-OH] is not a product of known phytases [33], including multiple inositol polyphosphate phosphatase, MINPP [34,35]. Because the other InsP_5_ species are only minor components of these tissues, the data shown (Figure 5) make it likely that a common ‘soluble’ pathway/network of InsP_6_ synthesis, Ins(3,4,6)P_3_ to Ins(3,4,5,6)P_4_ to Ins(1,3,4,5,6)P_5_ to Ins(1,2,3,4,5,6)P_6_ that is discrete from the lipid-derived pathway that contributes Ins(1,4,5)P_3_ to cytosolic InsP metabolism [32], is operative organ wide in avians. Moreover, the absence of the 2-phosphate distinguishes intermediates of synthesis (Figure 5E) from lumenal digestion (Supplementary Figure S1D).

While *myo*-inositol is the scaffold on which our understanding of biological function of InsPs is built in mammals, avians, fungi and plants, *myo*-inositol hexakisphosphate (InsP_6_) is not the only naturally biological isomer: apart from its presence with other isomers in soil [19,36], *neo*-inositol hexakisphosphate (*neo*-InsP_6_) is found in *Entamoeba histolytica* [37]. Use of LC-ICP-MS discounts the presence of D-*chiro*-, *neo* and *scyllo*-inositol hexakisphosphates in the avian tissues/organs analysed. This issue has not, to our understanding, been tested formally elsewhere.

### InsPs of duodenum, jejunum and ileum tissue are responsive to diet

A central theme of gut microbiome research is that the gut epithelium is responsive to factors generated in the gut lumen. While there are remarkably few studies of phytate digestion in rodent models, let alone humans, it was shown recently that oral gavage of mice, whose microbiota had been denuded by antibiotic treatment, with phytase producing microorganisms resulted in digestion of gavaged phytate, InsP_6_ [38]. Other than a recent report of application of CE-MS to mouse tissues including the colon and a single human biopsy thereof [39], and measurements of InsP_6_ and unspecified PP-InsPs by HILIC-MS/MS [40,41], we are not aware of detailed speciation of InsPs in gut tissues. Consequently, it is not known whether digestion of phytate has direct influence on gut InsP signalling or on broader mammalian physiology beyond mineral deficiency [5,6]. In contrast, for avian species, numerous feeding trials testing the effect of phytase treatment on InsP_6_ digestion and animal performance have been reported [reviewed 42]. They underpin practice in a food sector that raises approximately 70 billion chickens per annum. Nevertheless, the effect of InsP_6_ digestion on gut tissue InsPs is undefined in any organism. Previously, by *ad libitum* feeding of diets supplemented or not with phytase, we showed that InsP metabolism of kidney tissue of broiler chickens is responsive to diet, and to the interaction of inositol and phosphate released in the gut [28]. Remarkably, this premise has not been tested in other species but must mechanistically involve direct influence of diet on gut tissue, because gut tissues are the conduit by which digestion products enter the circulatory system.

**Figure 6:**
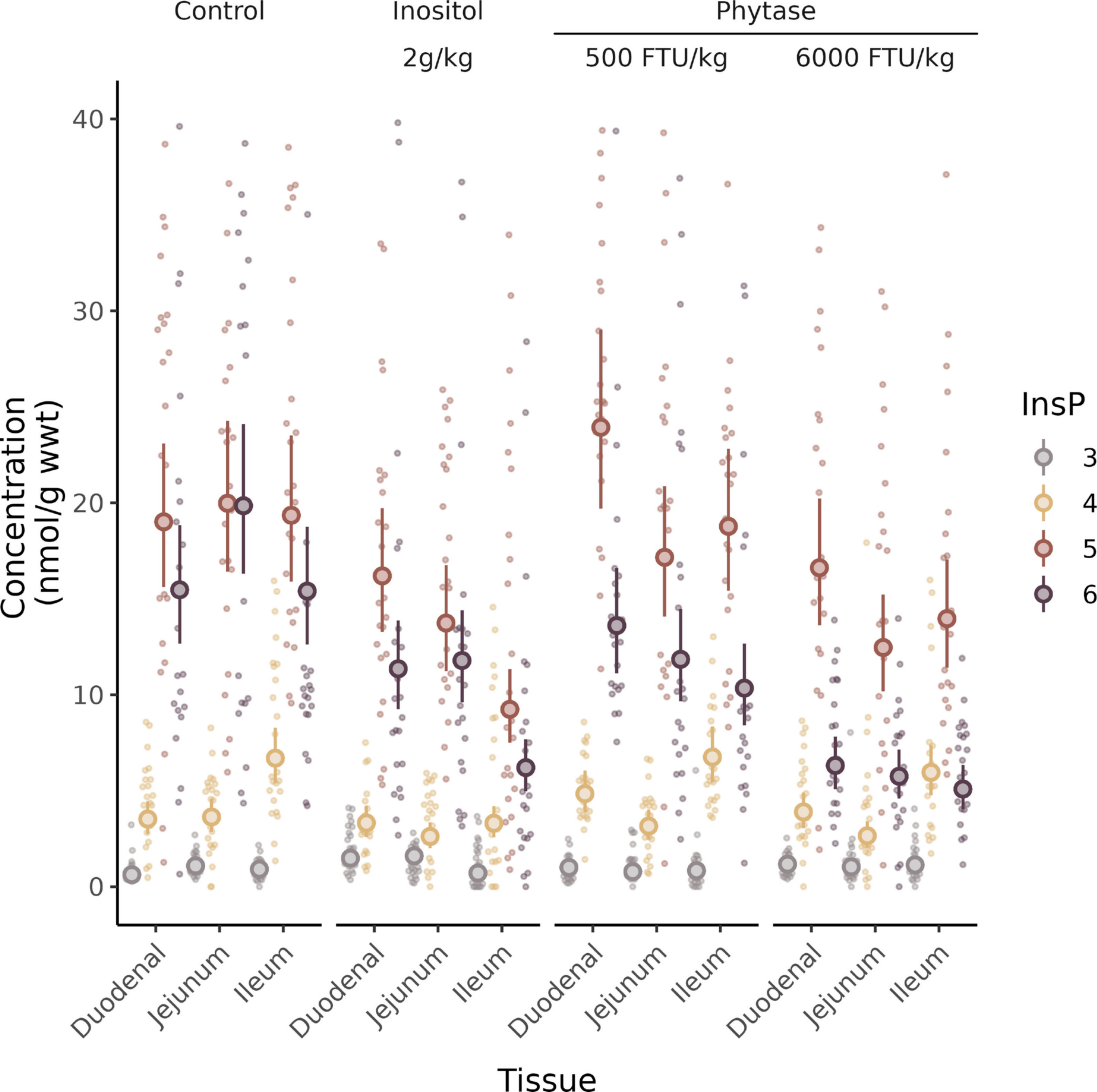
**Modulation of gut tissue InsPs by diet**. InsPs extracted from duodenum, jejunum and ileum tissues of broiler chickens fed different diets were analysed by HPLC. The diets comprise a control diet and the same supplemented with 2 g/kg *myo*-inositol or 500 or 6000 FTU/kg phytase. 2 g/kg inositol represents the amount of inositol that could be released in total from the InsP_6_ content of the control feed. Individual faded points represent individual InsP measures. Individual classes of InsP, viz. InsP_3_, InsP_4_, InsP_5_ and InsP_6_ are indicated 3, 4, 5 and 6 in the legend right. Large symbols and error bars represent estimated mean and 95% confidence intervals calculated by a general linear mixed model. The contrasts generated by the mixed model are shown in Supplementary Table S1.

To test whether gut tissue signalling molecules, InsPs, are responsive to diet, we performed an animal feeding trial. The absence of detectable PP-InsPs in the tissue samples analysed in Figure 5 allowed use of the less-sensitive but more accessible HPLC-UV method [23]. We note that the level of InsP_6_ measured in chicken gut tissues for control diet-fed animals, 15–20 nmol/g wet weight, is comparable with that measured approximately 70 nmol/g in mouse colon [39], and both are 2.5–10-fold less than reported by HILIC-MS/MS [41]. For mice, the authors suggested that PP-InsPs of gut tissues may have arisen from uptake from the gut lumen, i.e., been present in feed which was autoclaved. For our study, we have described the active phytase activity of the mash diet. It is possible therefore that the absence of PP-InsPs in chicken gut tissues may reflect their digestion by feed and adjunct phytases. Again, we know of no other measurement for this tissue/organ. The data presented in Figure 6 represent analysis of approximately 300 perchloric acid-extracted and TiO_2_-purified tissue samples. For each dietary treatment: control, 2 g/kg inositol, 500 or 6000 FTU phytase/kg, all with or without titanium dioxide, an inert marker of digestion, samples of tissue were taken from the duodenum, jejunum and ileum of 24 randomly selected individual birds (from a population of 480) and analysed by HPLC. The feeding trial design was reported [43]. Figure 6 shows the levels of InsP_3_, InsP_4_, InsP_5_ and InsP_6_ measured, besides the estimates of the mean and standard deviation generated by a linear mixed model of data set.

The data presented in Supplementary Table S1 show the differences between the mean slopes, their confidence intervals and the probabilities, referenced to the InsP_3_ value of duodenal tissue samples taken from birds fed a control diet lacking titanium dioxide. Post hoc analysis of the global data set, i.e., without stratification, shows that gut tissue InsPs were responsive to diet. The model predicts 75% of the variance across the entire data set. The jejunum showed a significant differential in response to the highest phytase levels between mean InsP levels (LMM post hoc: Control − Phytase 6000 FTU/kg *t_2270_* = −4.659 [nmol/g w wt], *P*<0.001). Ileum tissue showed a significantly differential increase in InsP to inositol (LMM post hoc: Control − Inositol *t_227_* = 5.593, *P*<0.001) and a marginally significant decrease in InsP to phytase (LMM *post hoc*: Control − Phytase 6000 FTU/kg *t_227_* = 3.267, *P*=0.007). By individual InsPs, there were no differential responses in InsP_3_, InsP_4_ or InsP_5_ to phytase levels (*P*>0.05) for any gut tissue, except for a marginally significant response for InsP_5_ in jejunum (LMM Post hoc: Control − Phytase 6000 FTU/kg: *t_227_* = 3.6, *P*=0.002). All gut tissues showed a significant change in InsP_6_ levels with the highest phytase levels (LMM *Post hoc*: Control − Phytase 6000 FTU/kg; duodenum *t_227_* = 6.59, *P*<0.001, ileum *t_3227_* = 8.04, *P*<0.001, jejunum *t_227_* = 9.16, *P*<0.001).

Because of the central role of InsP_6_ in higher InsP and PP-InsP synthesis and the singular route of InsP_6_ synthesis, from InsP_5_ [2-OH]^32^, we tested for effect of diet on InsP_5_:InsP_6_ ratio. Significant difference in InsP_5_ [2-OH]:InsP_6_ ratio was also detected between Control and Phytase 6000 groups for duodenum (*t_18.3_ = −*3.2*, P*=0.002) and jejunum (*t_19_ = −*4.7*, P*<0.001), but not for the ileum (t*_18.4_* = −2.357, *P*=0.12) (Supplementary Table S2). These data highlight how important signalling molecules of tissue of the gut–lumen interface, InsP_5_ [2-OH] and InsP_6_, are manipulable by diet with dietary effects likely targeting InsP_5_ 2-kinase. This enzyme is strongly reversible [44]. The results of these studies illustrate the utility of LC-UV and LC-ICP-MS for InsP analysis and particularly for gut-microbiome research. The data are also presented in Supplementary Tables S3, 4 and 5.

## LC-ICP-MS analysis of plant tissue

**Figure 7:**
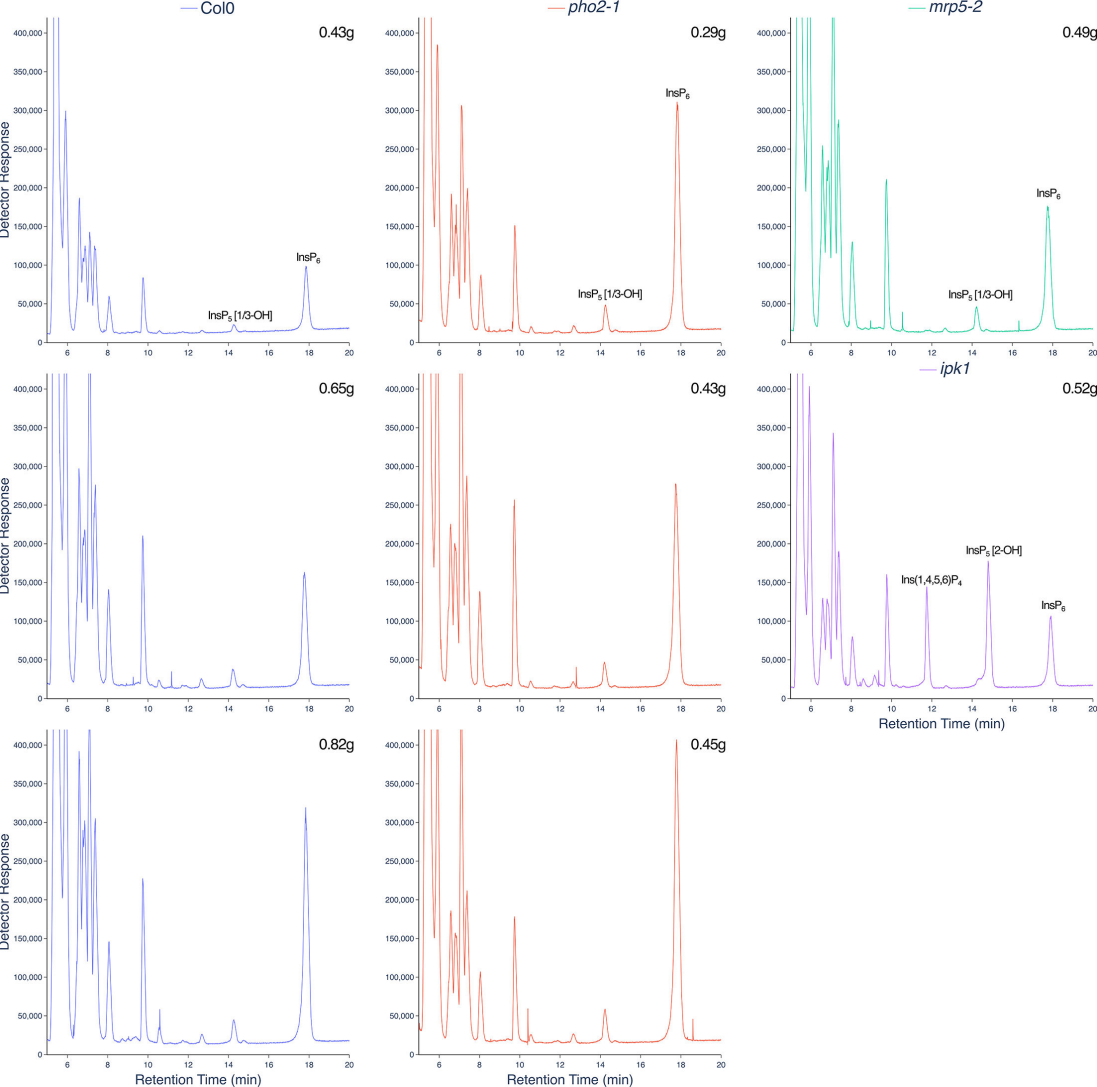
Inositol phosphate profiles of wildtype (Col0) and PSR mutants. Plants grown for 12 weeks on soil were weighed, frozen in liquid N_2_, ground and extracted with perchloric acid. The extract was applied to TiO_2_, recovered with NH_4_OH, lyophilized and recovered in 300 μl of water. A 42.5 μl aliquot was applied to LC-ICP-MS. The masses of the plants from which extracts were prepared are shown for each genotype: Col0 (blue), *pho2-1* (red), *mrp5-2* (green), *ipk1* (purple).

Phosphate is a major nutrient that limits plant growth. The mechanisms by which plants sense changing phosphate status of their tissue are intensely studied with predominant roles in the Phosphate Starvation Response identified for InsP kinases of the ITPK [45], IPK1 (IP_5_ 2 K) [46,47] and VIP (VIH) families [48], reviewed [49]. These garner interest beside transporters that integrate whole plant response to phosphate availability [50]. Replicate extractions of soil-grown Col0 and *pho2-1* [51] plants are shown (Figure 7). The latter shoot phosphate hyper-accumulation mutant which bears mutation in the *UBC24* gene [52] shows levels of InsP_6_ approximately double that of wildtype Col0, but without substantive change in overall InsP profile. *ipk1*, extensively characterized in terms of InsP profile and phosphate hyper-accumulation, shows elevations in Ins(3,4,5,6)P_4_ and InsP_5_ [2-OH] as well as substantial reduction in InsP_6_, whether measured by radiolabelling [45,47,53], CE-MS [53], or illustrated here for a single sample by LC-ICP-MS (Figure 7). The ABC transporter mutant *mrp5* [54] also shows reduced seed InsP_6_ and levels of InsPs like wildtype in vegetative tissues [54,55], shown here for a single sample (Figure 7).

## Discussion

Considering the contribution of InsPs and PP-InsPs to cellular processes, including phosphate homeostasis and energy status [3,46–49,55], we thought that it might be useful to construct a technique, with capability of measuring phosphorus content of multiple isomers of InsPs and PP-InsPs alike. The value of the measurement of other InsPs that are the metabolic precursors of InsP_5_ species, InsP_6_ and PP-InsPs, besides PP-InsPs themselves, is obvious not least because ‘small’ changes may be overlooked, certainly where PP-InsPs are normalized to InsP_6_, or where canonical pathways are assumed. More generally, without adoption of methods that cover the widest spectrum of InsPs, from InsP_1_ to InsP_6_ and PP-InsPs and that can distinguish species unique to different pathways, the pleiotropic effects of disruption of individual genes on broadest InsP metabolism are likely hidden – particularly, where analysis is restricted to PP-InsPs.

By demonstrating the utility of LC-ICP-MS to handle diverse extractants, we show how the approach is relevant to InsP measurement in environmental samples and across taxa – here in *Dictyostelium*, plants and animals – and most obviously in the context of animal nutrition and phosphate homeostasis. For the latter, we have shown how LC-ICP-MS is suitable for the study of InsPs in animal physiological contexts. The differences in InsPs of the gut lumen and gut tissues constitute transmembrane gradients of important bioactive species. While the extent of vectorial transport of native InsPs across gut epithelia has not been described but has been suggested for PP-InsPs in mice [39], exchange of the contents of extracellular vesicles of the gut microbiome, particularly of gram negatives such as *Bacteroidetes*, with gut epithelia is an emergent field [34,56]. Less ambiguously, InsP_6_ is transported subcellularly by the ABC transporter MRP5 (ABCC5) [54]. The presence of extracellular InsPs in kidney stones [57] and in the laminal layers of hydatid cysts of the *Echinococcus granulosus* [58] affords further evidence of InsP transport in metazoan taxa. We reveal common synthesis/metabolism of InsPs in duodenum, jejunum, ileum, erythrocyte and kidney of broiler chickens by a non-canonical pathway that is not obviously/directly related to PP-InsP turnover [30,32] and which, though isomerically distinct from digestion of dietary InsP_6_ is demonstrably connected thereto. We show a pronounced effect of diet on this non-canonical pathway in tissue of the duodenum, jejunum and ileum, as well as effect on kidney [28]. Put simply, InsP signalling molecules are shown to be responsive to diet at an interface, the gut, that has become the focus of human health research. Indeed, these tissues have a major role in immunity [59,60] as well as digestion.

In model systems, the effect of disruption of inositol phosphate multikinase (IPMK) has pleiotropic effects. These are attributed to InsPs and PP-InsPs [8,61] and include influence on intestinal function [14,62]. Interestingly, disruption of IP6K2 was shown to alter PP-InsP_5_ in rodent knock-out models, largely without effect on InsP_6_. The levels of InsP_6_, and consequently PP-InsP_5_, in mouse gut tissues: stomach, small intestine, duodenum and colon, measured by HILIC MS [40] were considerably higher (e.g. approaching 600 and 22 pmol per mg, respectively, for small intestine) than in other tissues [41]. The study did not describe isomers other than InsP_6_, but showed that purification of the diet which removed approximately 94% of InsP_6_ and PP-InsP_5_ altered gut tissue InsP_6_ and PP-InsP_5_ levels. The mechanism by which diet influenced tissue InsP and PP-InsP levels was not the subject of the study, but the level of InsP_6_ in the standard diet (approximately 3.96 nmol/mg) is approximately 21% of that used in the present study.

Against these findings, the current study highlights the role of diet in manipulation of gastro-intestinal tissue InsPs, and by extrapolation, most likely, PP-InsPs. Our study identifies an area of physiology ripe for investigation and, moreover, provides the tools by which a broad understanding of the InsP complement of animal tissues can be obtained without especial selection of subsets of InsP species.

## Methods and Materials

InsPs and PP-InsPs were obtained from sources described [23,24]. Among these, the InsP_6_ isomers were obtained from the soil-extracted collections of the late Dennis Cosgrove and Max Tate [cited in 63].

InsP_8_ (1,5-(PP)_2_-InsP_4_) and *rac*-InsP_8_ (racemic-1,5-(PP)_2_-InsP_4_) were synthesized using methodology developed for 5-PP-InsP_5_ [64], employing intermediates described in [65]. These were purified by ion-exchange chromatography and thoroughly characterized by NMR and mass spectrometry. They were>95% pure and were used as their triethylammonium salts.

## Extraction

Cells, tissues and organisms were extracted with cold 0.8 M HCl, 1 M HClO_4_ or 100 mM NaF, 20 mM EDTA pH 10, with further details in figure legends. Soil was extracted at a ratio of 1 g to 10 ml of 50 mM sodium EDTA, 250 mM NaOH [19]. Gut tissue extracts (100 mg frozen tissue) in HClO_4_ were pre-concentrated on TiO_2_ and recovered finally in 100 μl water [28].

## HPLC parameters for ICP-MS

Samples were injected from a 42.5 μl or 200 μl loop of Thermo ICS3000 autosampler. A Dionex IC3000 pump delivered gradients. Compounds, including InsPs and PP-InsPs, were resolved on a 3 mm × 250 mm Dionex CarboPac PA200 column, fitted with 3 mm × 50 mm guard column, by elution with HCl at a flow rate of 0.4 ml min^−1^. The column eluate was directed *in toto* to the PFA concentric nebuliser (Thermo Scientific) of a Thermo iCAP Tq (Thermo Scientific) inductively coupled plasma spectrometer. Two gradient forms were used: either linear or exponential. For the linear gradient, the solvents A (water) or B (0.8 M HCl) were mixed according to the following schedule: time, min; % B; 0, 0; 25, 100; 38, 100; 39, 0; 40, 0. For the exponential gradient, a gradient function ‘7’ was applied in the Chromeleon v.7 software of the Dionex HPLC system during the 0–25 min interval of the same gradient.

## ICP-MS

The ICP-MS machine was controlled using Thermo Qtegra (Thermo Scientific) software with instrument parameters for phosphorus detection as described [19]. The machine was operated in collision mode whereby the first quadrupole was set at m/z 31^+^; for P, the second quadrupole provided a reaction cell for reaction with oxygen, generating a PO^+^ ion m/z 47^+^ that was filtered in the third quadrupole. The detector’s dwell time was set to 50 msec or 500 msec, giving up to 45,000 data points for a 45 minute run.

## HPLC-UV

Samples, 50 μl for tissue, were injected from a 200 μl loop of a Jasco (Japan) LC-4000 HPLC system comprised of a AS-4195 autosampler, a Jasco PU-4185 gradient pump and a UV-4075 detector set at 290 nm. As for LC-ICP-MS, InsPs were resolved on a 3 mm × 250 mm Dionex CarboPac PA200 column, fitted with 3 mm × 50 mm guard column. The column was eluted with either HCl or methanesulphonic acid at a flow rate of 0.4 ml min^−1^. The column eluate was mixed with 0.1% w/v Fe(NO_3_)_3_.9H_2_O in 2% w/w HClO_4_ [66]. This was delivered post-column into a mixing Tee and from there to a 190 μl volume, 0.25 mm internal diameter reaction coil (delivered by a Jasco PU-4085 pump at a flow rate of 0.2 ml min^−1^) before passage to the UV detector. Linear gradients of solvents: A (water) or B (HCl or methanesulfonic acid) were mixed according to the following schedule: time, min; % B; 0, 0; 25, 100; 38, 100; 39, 0; 40, 0. Identification of InsPs was made by comparison with a reference sample of InsPs prepared by acid-hydrolysis of InsP_6_ [67]. Concentration of InsPs was established by reference to UV detector response to injection of InsP_6_ [23]. Example coefficients of variation for retention time of Ins(3,4,5,6)P_4_, Ins(1,3,4,5,6)P_5_ and InsP_6_, with mean retention times: 19.37, 26.37 and 33.66 min, respectively, were 1.008, 0.959 and 1.133% for a set of 72 ileal tissue samples run with standards over a period of three days.

## Data processing

LC-ICP-MS data were exported from Chromeleon software as *x*,*y* data (.csv) and imported into Jasco ChromNav v.2 software for peak integration. For graphical presentation of chromatograms, *x*,*y* data were imported into and plotted in ggplot2 after smoothing with a Savitzky–Golay filter with window length of 11 and polynomial order of 2.

LC data generated during measurement of InsPs by post-column addition of ferric nitrate were exported from Jasco ChromNav v.2 software as *x*,*y* data (.csv) [24]. Data were imported into and plotted in ggplot2 without smoothing or mathematical manipulation.

## Statistical analysis

For the animal feeding trial, the results of which are shown in Figure 6, all analyses were carried out in R ver 4.3.1 [68], with input the InsP content of tissues calculated from the integrated HPLC-UV traces; examples of which are shown for digesta in Supplementary Figure 1. Linear mixed-effects models (LMM) were fitted with the lmerTest package [69], summarised with emmeans [70] and model residuals were checked for violations of assumptions with the DHARMa package [71]. Figures were generated with ggplot2. Data analyses are briefly summarised below.

For individual InsP values, we fitted a linear mixed model on log-transformed values with a constant (1) added to each value, with diet (control, inositol added and two levels of phytase supplementation), InsP type, titanium addition and tissue type as fixed effects, and two-way interactions between InsP type, tissue and diet, the model also included individual and dietary pen as random effects. When comparing the InsP_5_:InsP_6_ ratio, we modelled a square root transformation of the ratios with diet, tissue type and titanium addition included as fixed effects, with an interaction between diet and tissue type and individual included as random effect. The model also included individual and dietary pen as random effects.

All linear mixed-effect models were fitted with REML and the nloptwrap optimiser for model convergence. Where appropriate, degrees of freedom were estimated with Satterthwaite’s approximation. Post hoc pairwise comparisons were carried out with the emmeans package and a Tukey adjustment for multiple comparisons.

## Animals, diet and experimental design

Birds, 480 male Ross 308 hatchlings, obtained from a commercial hatchery (PD Hook, Cote, Oxford, UK) were allocated randomly to 48 floor pens on day 1. Animals were divided among eight treatment (diet) groups (Supplementary Table S6). Of these diets, half were supplemented with 5 g/kg TiO_2_ (Titanium, Ti, a common inert marker of digestion) and the other half were not. With/without Ti, diets were labelled as Control (no further supplementation), Ins (supplemented with 2 g/kg ^12^C/^13^C inositol, containing ^13^C inositol at d30‰), or Phy500 or Phy600 (supplemented with 500 or 6000 FTU/kg phytase). The phytase used was Quantum Blue and was supplied by AB Vista (Marlborough, UK). The composition of the basal diet, see [43], was formulated according to the Ross Management Manual 2018. Ten birds were allocated to six replicate pens for each treatment group with birds fed the respective diets throughout the trial (1–21 days). A power calculation was made using data for response of mean gizzard and ileal inositol contents to phytase addition [72], indicating that six replicates per treatment were sufficient to identify treatment differences at a power setting of 80% and a type 1 error rate of 5%. Birds had *ad libitum* access to feed and water throughout the study.

## Sampling

Birds, two per pen, were selected at random and euthanised on d 21 post-hatch by cervical dislocation without prior stunning in accordance with the Welfare of Animals at the Time of Killing (England) Regulations (2015) guidelines for poultry. For each bird, the gizzard was excised, opened and the contents scraped into a container as a pooled sample from both birds. Ileal digesta were collected from the same two birds by gentle digital pressure, pooled and stored at −20°C prior to lyophilization. They were subsequently freeze-dried at −50°C for seven days or until constant weight. Once dried, samples were finely ground using a coffee grinder and stored at 4°C until analysis.

From each of the two birds from which digesta was pooled for analysis, duodenum, jejunum and ileum samples were excised, taking care to ensure tissue was consistently excised from the same region of organ for each bird. Samples were immediately frozen at −20°C before shipping to UEA and thereafter were stored at −80°C. After defrosting, 100 mg slices of tissue were taken for InsP extraction and analysis.

## Analysis of InsPs in digesta

Diets, gizzard and ileal digesta were extracted as described [43]. In brief, 100 mg samples of milled, dry feed or digesta were extracted in 5 ml of 100 mM NaF, 20 mM Na_2_EDTA (pH 10) for 30 minutes shaking, followed by 30 minutes in a chilled bath sonicator and a further 2 hours standing at 4°C. The extract was centrifuged at 9000×g for 15 minutes at 4°C and 1 ml was filtered through a 13 mm 0.45 μm PTFE syringe filter (Kinesis, UK). Aliquots (20 μl) were analysed by HPLC with UV detection at 290 nm after post-column complexation of InsPs with ferric ion.

## Analysis of InsPs in gut tissues

Tissue (100 mg frozen weight) was homogenised with an Ultra-Turrax (IKA T-10 Ultra-Turrax® High-Speed Homogeniser) with 8 mm stainless steel probe (S 10 N - 8 G ST) in 600 μl 1M HClO_4_ in a Pyrex glass tube on ice. After transfer to 1.5 ml tubes, the samples were held on ice for 20 minutes with vortexing at 10 minute intervals and centrifuged at 13,000×g for 10 minutes at 4°C. Following removal of an aliquot (20 μl) which was diluted to 1000 μl with 18.2 Megohm.cm water for analysis of inositol, the cleared lysates were applied to titanium dioxide (TiO_2_) beads (Titansphere® TiO_2_ 5 μM, Hichrom) [73].

## Analysis of InsPs recovered from TiO_2_

Perchloric acid extracts in their entirety, minus the aliquot taken for inositol analysis, were applied to 5 mg of titanium dioxide (TiO_2_) beads (Titansphere® TiO_2_ 5 μM, Hichrom) and incubated for 30 minutes with mixing on a rotator. Thereafter, samples were centrifuged at 3500×g for 5 minutes and the HClO_4_ supernatant was discarded. InsPs were eluted from beads resuspended in 200 μl 3% ammonium hydroxide solution (pH 10), with vortexing and incubation for 5 minutes at 4°C. After centrifugation, 3500×g for 1 minute, the supernatant was transferred to a clean 1.5 ml tube and the beads were further extracted with a second 200 μl aliquot of ammonium hydroxide [73]. The eluates were pooled, freeze-dried until dry and resuspended in 100 μl of 18.2 MOhm cm water for further analysis by HPLC.

## Supplementary material

Online supplementary material 1

## Data Availability

Data associated with Figure 6 are available at https://research-portal.uea.ac.uk/en/persons/charles-brearley/datasets/

## Abbreviations

EDTA: Ethylenediamine tetra-acetic acid
HEPES: 4-(2-hydroxyethyl)-1-piperazineethane sulfonic acid
HILIC: Hydrophilic Interaction Liquid Chromatography
HPLC: High-pressure liquid chromatography
His: Histidine
IPK1: Inositol pentakisphosphate 2-kinase
IP_5_K: Inositol pentakisphosphate 2-kinase
IP_6_K: Inositol hexakisphosphate kinase
ITPK: Inositol tris/tetrakisphosphate kinase
InsP_6_: Ins(1,2,3,4,5,6)P_6_, *myo*-inositol 1,2,3,4,5,6-hexakisphosphate
InsP_8_: Bis-diphospho-*myo*-inositol-tetrakisphosphate
1,5-InsP_8_: 1d-1,5-bis-diphospho-*myo*-inositol 2,3,4,6-tetrakisphosphate
3,5-InsP_8_: 1d-3,5-bis-diphospho-*myo*-inositol 1,2,4,6-tetrakisphosphate
4,5-InsP_8_: 1d-4,5-bis-diphospho-*myo*-inositol 1,2,3,6-tetrakisphosphate
6,5-InsP_8_: 1d-6,5-bis-diphospho-myo-inositol 1,2,3,4-tetrakisphosphate
Ins(1,2,3,4)P_4_: 1d-myo-inositol 1,2,3,4-tetrakisphosphate
Ins(1,2,3,5)P_4_: *myo*-inositol 1,2,3,5-tetrakisphosphate
Ins(1,3,4,5)P_4_: 1d-*myo*-inositol 1,3,4,5-tetrakisphosphate
Ins(1,4,5,6)P_4_: 1d-*myo*-inositol 1,4,5,6-tetrakisphosphate
Ins(2,3,4,5)P_4_: 1d-*myo*-inositol 2,3,4,5-tetrakisphosphate
Ins(2,3,4,6)P_4_: 1d-*myo*-inositol 2,3,4,6-tetrakisphosphate
Ins(2,4,5,6)P_4_: *myo*-inositol 2,4,5,6-tetrakisphosphate
Ins(3,4,5,6)P_4_: 1d-*myo*-inositol 3,4,5,6-tetrakisphosphate
Ins(3,4,5,6)P_4_: 1d-*myo*-inositol 3,4,5,6-tetrakisphosphate
InsP_5_ [1-OH]: Ins(2,3,4,5,6)P_5_, 1d-*myo*-inositol 2,3,4,5,6-pentakisphosphate
InsP_5_ [3-OH]: Ins(1,2,4,5,6)P_5_, 1d-*myo*-inositol 1,2,4,5,6-pentakisphosphate
InsP_5_ [4-OH]: Ins(1,2,3,5,6)P_5_, 1d-*myo*-inositol 1,2,3,5,6-pentakisphosphate
InsP_5_ [5-OH]: Ins(1,2,3,4,6)P_5_, *myo*-inositol 1,2,3,4,6-pentakisphosphate
InsP_5_ [6-OH]: Ins(1,2,3,4,5)P_5_, 1d-*myo*-inositol 1,2,3,4,5-pentakisphosphate
1-InsP_7_(1-PP-InsP_5_): 1d-1-diphospho-*myo*-inositol 2,3,4,5,6-pentakisphosphate
2-InsP_7_(2-PP-InsP_5_): 2-diphospho-*myo*-inositol 1,3,4,5,6-pentakisphosphate
3-InsP_7_(3-PP-InsP_5_): 1d-3-diphospho-*myo*-inositol 1,2,4,5,6-pentakisphosphate
4-InsP_7_(4-PP-InsP_5_): 1d-4-diphospho-*myo*-inositol 1,3,5,6-pentakisphosphate
5-InsP_7_(5-PP-InsP_5_): 5-diphospho-*myo*-inositol 1,2,3,4,6-pentakisphosphate
6-InsP_7_(6-PP-InsP_5_): 1d-6-diphospho-*myo*-inositol 1,2,3,4,5-pentakisphosphate
3-PP-Ins(1,2,4,5)P_4_: 1d-3-diphospho-*myo*-inositol 1,2,4,5-tetrakisphosphate
5-PP-Ins(1,2,3,4)P_4_: 1d-5-diphospho-*myo*-inositol 1,2,3,4-tetrakisphosphate
VIH: Arabidopsis thaliana diphosphoinositol pentakisphosphate
*neo*-InsP_6_: *Neo*-inositol 1,2,3,4,5,6-hexakisphosphate
rac-InsP_8_: 1:1 mixture of 1,5-InsP_8_ and 3,5-InsP_8_
*scyllo*-InsP_6_: *Scyllo*-inositol 1,2,3,4,5,6-hexakisphosphate
