# Supplementary material for: Phosphorus-specific, liquid chromatography inductively coupled plasma mass spectrometry for analysis of inositol phosphate and inositol pyrophosphate metabolism
Source: Biochem J. 2025 Nov 4;482(21):1627–44. doi: 10.1042/BCJ20253151 (PMC7618242; doi:10.1042/BCJ20253151)
Supplement: Online supplementary material 1 [file bcj-482-21-BCJ20253151-s001.docx]

**Supplementary Information**

**Phosphorus-specific, liquid chromatography inductively coupled plasma mass-spectrometry**

**for analysis of inositol phosphate and inositol pyrophosphate metabolism**

^1^Colleen Sprigg, ^1^Hayley Whitfield, ^1^Philip T. Leftwich, ^2^Hui-Fen Kuo, ^2^Tzyy-Jen Chiou, ^3^Adolfo Saiardi, ^4^Megan L. Shipton, ^4^Andrew M. Riley, ^4^Barry V.L. Potter, ^5^Dawn Scholey, ^5^Emily Burton, ^6^Mike R. Bedford, ^1^Charles A. Brearley

^1^School of Biological Sciences, University of East Anglia, Norwich Research Park, Norwich NR4 7TJ, UK;

^2^Agricultural Biotechnology Research Center, Academia Sinica, Taipei 115, Taiwan;

^3^Laboratory for Molecular Cell Biology, University College London, London WC1E 6BT, UK;

^4^Medicinal Chemistry & Drug Discovery, Department of Pharmacology, University of Oxford, Mansfield Road, Oxford OX1 3QT, UK;

^5^School of Animal, Rural and Environmental Sciences, Nottingham Trent University, Southwell, Nottingham NG25 0QF, UK;

^6^AB Vista, Marlborough SN8 4AN, UK

Michael R. Bedford orcid.org/0000-0002-5308-4290

Charles A. Brearley; orcid.org/0000- 0001-6179-9109

Emily Burton; orcid.org/0000-0003-2784-6922

Tzyy-Jen Chiou; orcid.org/0000-0001-5953-4144

Philip T. Leftwich; orcid.org/0000-0001-9500-6592

Barry V.L. Potter; orcid.org/0000-0003-3255-9135

Andrew M. Riley; orcid.org/0000-0001-9003-3540

Adolfo Saiardi; orcid.org/0000-0002-4351-0081

Megan L. Shipton; orcid.org/0000-0002-9982-0927

Colleen Sprigg; orcid.org/0000-0001-7755-4245

Hayley L. Whitfield; orcid.org/0000-0001-6003-7874

Figure S1. LC-ICP-MS analysis of InsP_6_ digestion in the avian gastrointestinal tract.

Figure S2. Biological variability and reproducibility of LC-ICP-MS

Table S1. Statistical analysis of phytase inclusion in diet on inositol phosphate content of gut tissues

Table S2. Analysis of effect of phytase inclusion in diet on InsP_5_: InsP_6_ ratio of gut tissues

Table S3. Inositol phosphate levels in duodenal segments of chicken

Table S4. Inositol phosphate levels in jejunum segments of chicken

Table S5. Inositol phosphate levels in ileum segments of chicken

Table S6. Dietary treatments and Test Substance inclusion rates


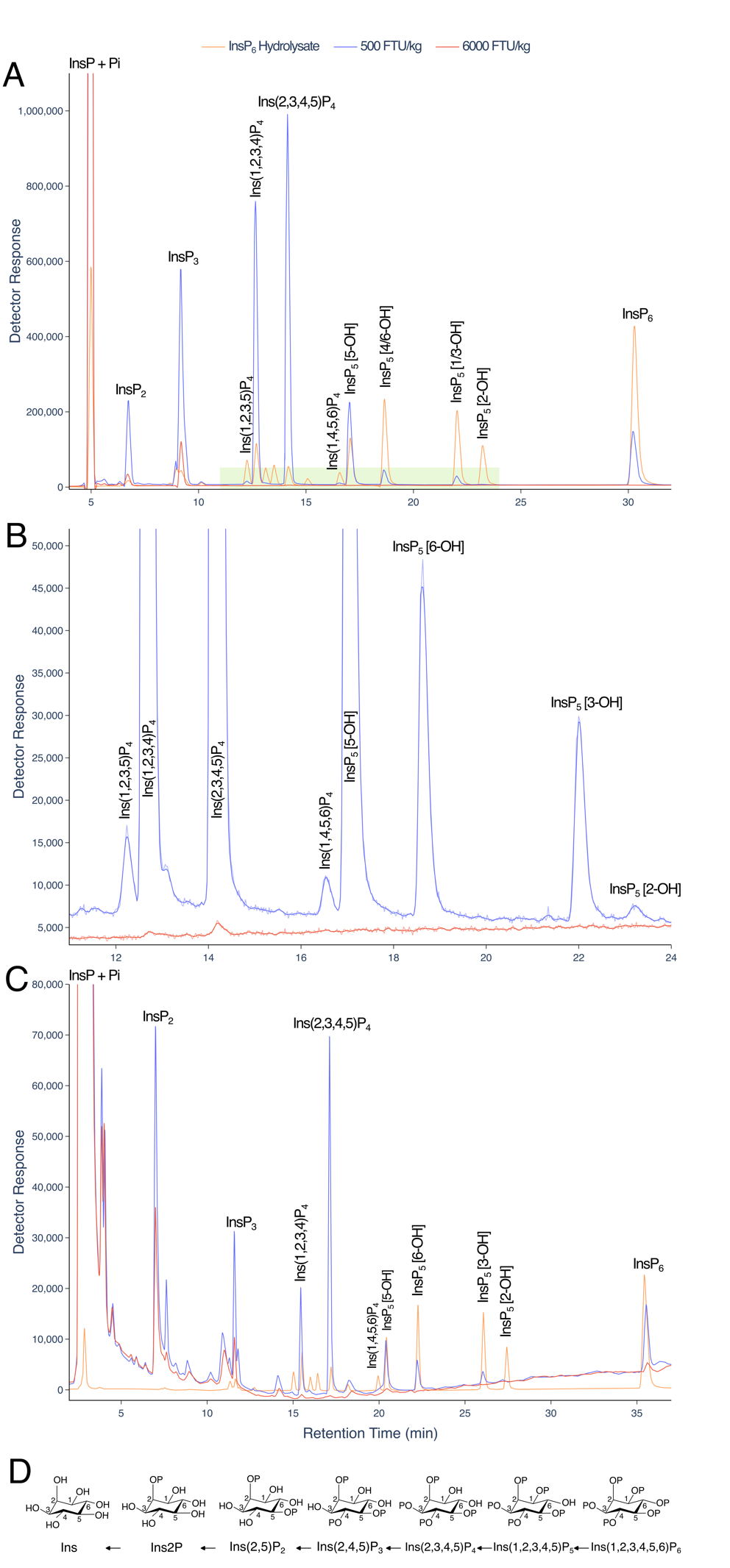


**Figure S1.** **LC-ICP-MS analysis of InsP_6_ digestion in the avian gastrointestinal tract**. **A**. LC-ICP-MS of lumenal gizzard content of birds fed a diet containing low (500 FTU/kg) (blue) or high (6000 FTU/kg) (red) phytase, beside a hydrolysate of InsP_6_ (orange). **B**. An expansion of the InsP_4_ and InsP_5_ region, indicated by the pale green panel, of the 500 FTU/kg and 6000 FTU/kg traces shown A. **C.** LC-UV analysis of lumenal content of gizzard of birds fed a diet containing low (500 FTU/kg) (blue) or high (6000 FTU/kg) (red) phytase, beside a hydrolysate of InsP_6_ (orange). **D**. Pathway of degradation of InsP_6_ by 6-phytase after ^1,2,3^. Final digestive dephosphorylation of InsP is likely catalysed by alkaline phosphatase of the mucosal epithelia. For all panels, samples were resolved on a CarboPac PA200 column eluted with methanesulfonic acid. Separations of gut lumen inositol phosphates matching the resolution shown (A, B, C) have been observed on more than 1000 occasions at varying phytase dose on CarboPac PA200 coupled to LC-UV. For the ICP analysis shown, various digesta samples have been analysed on more than 10 occasions with equivalent resolution and detector response.

**
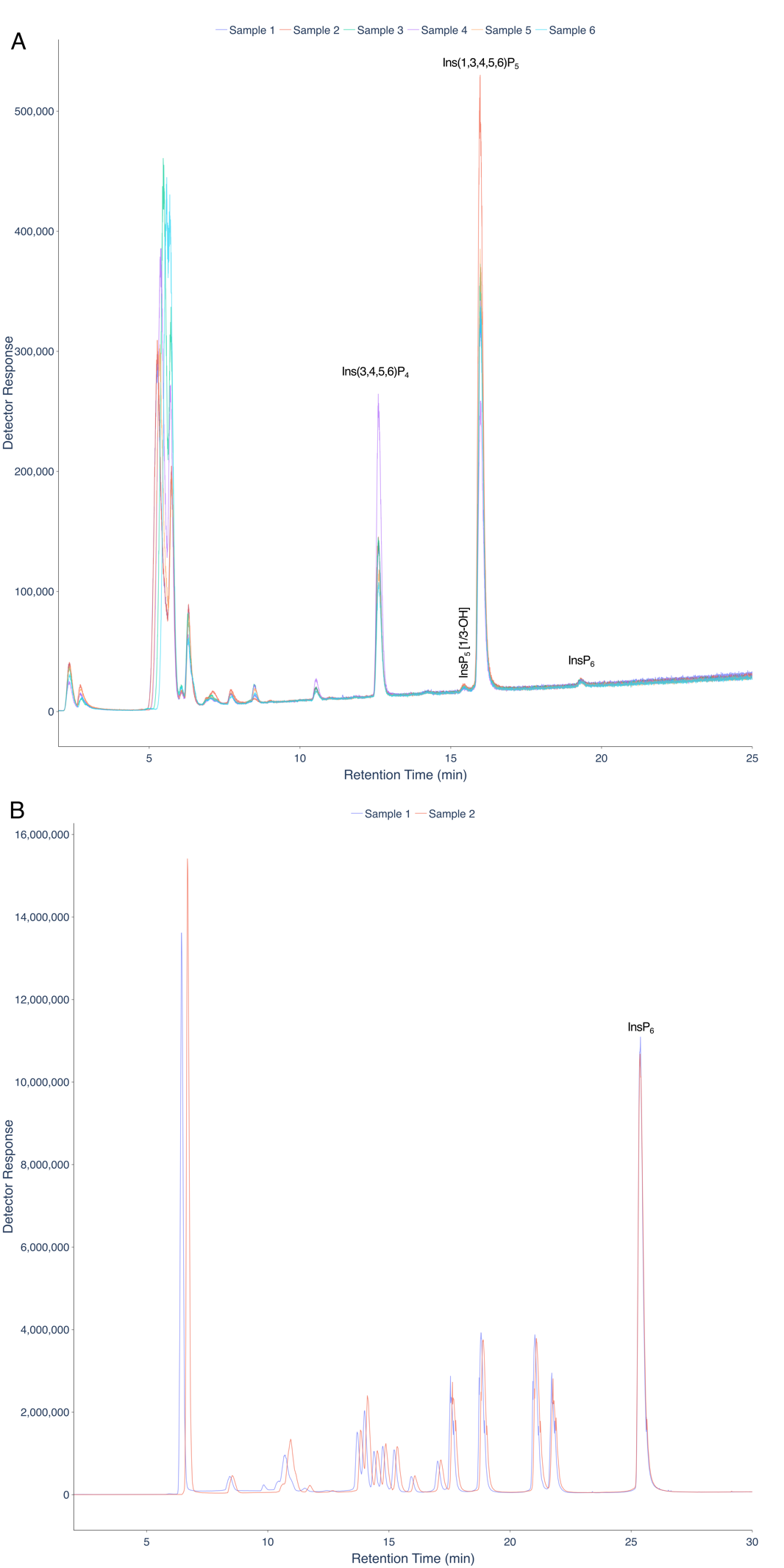
**

**Figure S2**. **Biological variability and reproducibility of LC-ICP-MS**. Inositol phosphate extracts were prepared from blood samples obtained from six 35 d old chickens raised in different pens and fed a control diet. Whole blood was extracted in HClO_4_, diluted with NaF-EDTA and analysed by LC-ICP-MS. The samples were analysed at the end of a set of 25 consecutive injections. For Ins(3,4,5,6)P_4_ and Ins(1,3,4,5,6)P_5_, with mean retention time (and coefficient of variation) 12.06 min (0.070) and 15.987 min (0.011), the two peaks had areas with mean (and standard error) of 1,516,234 (244,855) and 4,191,724 (425,478), respectively. B) Two replicate injections of an InsP_6_ hydrolysate sample were analysed at the end of a set of more than 50 consecutive injections. For these, the InsP_6_ peak area (counts.min) were 161,430,343 and 171,113,2621. The InsP_5_ peaks (not labelled), similarly, differed by less than 4% of the mean value for the duplicate measurements. In a separate experiment, three replicate injections of a different InsP_6_ hydrolysate sample gave peak areas (counts.min) for InsP_6_ of mean 2,041,016 and standard error 23,460. Different HCl gradients were used for A and B.

**Table S1. Analysis of effect of phytase inclusion in diet on inositol phosphate content of duodenum, jejunum and ileum tissue, analysed by a linear mixed-effects model with a log transformation (+1) applied to InsP measures.** Phytase was added, or not, at 500 or 6000 FTU/kg. Inositol was added at 2g/kg.

|  | | **log(value + 1)** | | | | | | |
| --- | --- | --- | --- | --- | --- | --- | --- | --- |
| *Predictors* | | *Estimates* | *CI* | | *p* |  |  |  |
| (Intercept) | | 0.43 | | 0.24 – 0.62 | **<0.001** | |  |  |
| Control [Inositol_added] | | 0.42 | | 0.18 – 0.66 | **0.001** | |  |  |
| Control [Phytase 500] | | 0.21 | | -0.03 – 0.45 | 0.092 | |  |  |
| Control [Phytase 6000] | | 0.29 | | 0.05 – 0.53 | **0.019** | |  |  |
| InsP_4_ | | 1.02 | | 0.82 – 1.23 | **<0.001** | |  |  |
| InsP_5_ | | 2.51 | | 2.30 – 2.71 | **<0.001** | |  |  |
| InsP_6_ | | 2.31 | | 2.11 – 2.52 | **<0.001** | |  |  |
| Tissue [Jejunum] | | 0.25 | | 0.06 – 0.44 | **0.011** | |  |  |
| Tissue [Ileum] | | 0.16 | | -0.03 – 0.35 | 0.098 | |  |  |
| Titanium [Yes] | | 0.11 | | 0.00 – 0.22 | **0.041** | |  |  |
| Control [Inositol added] × InsP_4_ | | -0.47 | | -0.71 – -0.23 | **<0.001** | |  |  |
| Control [Phytase 500] × InsP_4_ | | 0.05 | | -0.19 – 0.29 | 0.687 | |  |  |
| Control [Phytase 6000] × InsP_4_ | | -0.21 | | -0.45 – 0.03 | 0.081 | |  |  |
| Control [Inositol added] × InsP_5_ | | -0.57 | | -0.81 – -0.34 | **<0.001** | |  |  |
| Control [Phytase 500] × InsP_5_ | | 0.01 | | -0.22 – 0.25 | 0.918 | |  |  |
| Control [Phytase 6000] × InsP_5_ | | -0.42 | | -0.65 – -0.18 | **0.001** | |  |  |
| Control [Inositol added] × InsP_6_ | | -0.71 | | -0.95 – -0.47 | **<0.001** | |  |  |
| Control [Phytase 500] × InsP_6_ | | -0.33 | | -0.56 – -0.09 | **0.007** | |  |  |
| Control [Phytase 6000] × InsP_6_ | | -1.10 | | -1.34 – -0.86 | **<0.001** | |  |  |
| InsP_4_ × Tissue [Jejunum] | | -0.23 | | -0.43 – -0.02 | **0.031** | |  |  |
| InsP_5_ × Tissue [Jejunum] | | -0.20 | | -0.41 – 0.00 | 0.054 | |  |  |
| InsP_6_ × Tissue [Jejunum] | | -0.01 | | -0.22 – 0.19 | 0.899 | |  |  |
| InsP_4_ × Tissue [Ileum] | | 0.37 | | 0.16 – 0.58 | **<0.001** | |  |  |
| InsP_5_ × Tissue [Ileum] | | -0.14 | | -0.35 – 0.06 | 0.166 | |  |  |
| InsP_6_ × Tissue [Ileum] | | -0.17 | | -0.37 – 0.04 | 0.113 | |  |  |
| Control [Inositol added] × Tissue [Jejunum] | | -0.20 | | -0.41 – 0.00 | 0.054 | |  |  |
| Control [Phytase 500] × Tissue [Jejunum] | | -0.36 | | -0.57 – -0.16 | **0.001** | |  |  |
| Control [Phytase 6000] × Tissue [Jejunum] | | -0.32 | | -0.52 – -0.11 | **0.003** | |  |  |
| Control [Inositol added] × Tissue [Ileum] | | -0.54 | | -0.74 – -0.33 | **<0.001** | |  |  |
| Control [Phytase 500] × Tissue [Ileum] | | -0.25 | | -0.45 – -0.04 | **0.018** | |  |  |
| Control [Phytase 6000] × Tissue [Ileum] | | -0.18 | | -0.39 – 0.03 | 0.086 | |  |  |
| **Random Effects** | | | | | | |  |  |
| σ^2^ | | 0.26 | | | | |  |  |
| τ_00_ _id_ | | 0.05 | | | | |  |  |
| ICC | | 0.16 | | | | |  |  |
| N _id_ | | 96 | | | | |  |  |
| Observations | | 1152 | | | | |  |  |
| Marginal R^2^ / Conditional R^2^ | | 0.705 / 0.753 | | | | |  |  |

**Table S2. Analysis of effect of phytase inclusion in diet on InsP_5_: InsP_6_ ratio of duodenum, jejunum and ileum tissue, analysed by a linear mixed-effects model with a square root transformation applied to ratios.** Phytase was added, or not, at 500 or 6000 FTU/kg. Inositol was added at 2g/kg.

|  | **sqrt(InsP_5_: InsP_6_)** | | | |
| --- | --- | --- | --- | --- |
| *Predictors* | *Estimates* | *CI* | *p* | |
| (Intercept) | 1.28 | 1.10 – 1.47 | **<0.001** | |
| Control [Inositol added] | -0.01 | -0.26 – 0.23 | 0.918 | |
| Control [Phytase 500] | 0.17 | -0.07 – 0.42 | 0.169 | |
| Control [Phytase 6000] | 0.40 | 0.15 – 0.65 | **0.002** | |
| Tissue [Jejunum] | -0.21 | -0.45 – 0.02 | 0.076 | |
| Tissue [Ileum] | 0.12 | -0.12 – 0.35 | 0.335 | |
| Titanium [Yes] | -0.10 | -0.22 – 0.01 | 0.062 | |
| Control [Inositol added] × Tissue [Jejunum] | 0.24 | -0.10 – 0.57 | 0.164 | |
| Control [Phytase 500] × Tissue [Jejunum] | 0.14 | -0.19 – 0.48 | 0.395 | |
| Control [Phytase 6000] × Tissue [Jejunum] | 0.20 | -0.13 – 0.53 | 0.240 | |
| Control [Inositol added] × Tissue [Ileum] | 0.02 | -0.31 – 0.36 | 0.897 | |
| Control [Phytase 500] × Tissue [Ileum] | -0.10 | -0.44 – 0.23 | 0.541 | |
| Control [Phytase 6000] × Tissue [Ileum] | -0.10 | -0.44 – 0.23 | 0.536 | |
| **Random Effects** | | | |  |
| σ^2^ | 0.17 | | |  |
| τ_00_ _id_ | 0.02 | | |  |
| τ_00_ _Diet_ | 0.00 | | |  |
| N _id_ | 96 | | |  |
| N _Diet_ | 8 | | |  |
| Observations | 286 | | |  |
| Marginal R^2^ / Conditional R^2^ | 0.173 / NA | | |  |

Table S3. Inositol phosphate levels (nmol/g wwt) in duodenal segments of day 21 broilers

| Diet | InsP_3_ | InsP_4_ | InsP_5_ | InsP_6_ | ∑InsP |
| --- | --- | --- | --- | --- | --- |
| Control | 0.6±0.1^b^ | 4.1±0.7 | 21.2±4.2 | 14.4±3.9 | 40.3±8.4 |
| 2g/kg inositol | 1.1±0.2^ab^ | 2.6±0.5 | 12.0±1.2 | 13.5±4.7 | 29.2±5.2 |
| Phy500 | 0.8±0.1^ab^ | 5.0±0.6 | 26.1±2.7 | 13.0±1.0 | 44.9±3.3 |
| Phy6000 | 1.2±0.1^ab^ | 5.8±0.5 | 26.7±2.2 | 9.2±0.9 | 42.9±3.1 |
| Control TiO_2_ | 0.9±0.2^ab^ | 4.6±0.5 | 24.1±2.6 | 22.7±4.2 | 52.2±3.6 |
| 2g/kg inositol TiO_2_ | 2.8±0.2^aa^ | 4.0±0.5 | 21.7±2.3 | 21.1±6.7 | 49.7±8.1 |
| Phy500 TiO_2_ | 1.1±0.2^ab^ | 5.3±0.4 | 27.9±1.8 | 16.5±2.6 | 50.7±4.2 |
| Phy6000 TiO_2_ | 1.1±0.2^ab^ | 3.0±0.6 | 12.4±1.6 | 13.5±0.5 | 21.8±2.1 |

Data are given as group means ± SEM, n=12 (6 pens per diet with samples from 2 broilers per pen per treatment). Statistical analysis was performed by multiple T-tests with correction for multiple comparisons using the Holm-Šidák method. Differences in superscripts within columns indicate differences, at p < 0.05, between groups.

Table S4. Inositol phosphate levels (nmol/g wwt) in jejunum segments of day 21 broilers

| Diet | InsP_3_ | InsP_4_ | InsP_5_ | InsP_6_ | ∑InsP |
| --- | --- | --- | --- | --- | --- |
| Control | 1.2±0.1 | 3.8±0.6 | 19.5±2.3 | 33.5±8.9^a^ | 58.0±9.8^a^ |
| 2g/kg inositol | 0.8±0.2 | 2.2±0.5 | 12.6±1.6 | 20.9±8.0^ab^ | 36.4±8.2^ab^ |
| Phy500 | 1.4±0.3 | 4.1±0.4 | 22.3±3.2 | 11.4±2.5^b^ | 39.2±4.6^ab^ |
| Phy6000 | 1.8±0.6 | 5.0 ±1.3 | 15.7±2.1 | 5.8±0.8^b^ | 28.3±2.8^b^ |
| Control TiO_2_ | 1.2±0.2 | 3.8±0.6 | 22.6±3.1 | 29.7±6.0^ab^ | 57.3±6.7^ab^ |
| 2g/kg inositol TiO_2_ | 1.7±0.2 | 4.5±0.3 | 20.2±1.4 | 14.8±3.2^ab^ | 41.2±3.8^ab^ |
| Phy500 TiO_2_ | 0.9±0.2 | 2.7±0.5 | 16.1±1.9 | 16.2±3.0^ab^ | 25.9±4.2^ab^ |
| Phy6000 TiO_2_ | 1.0±0.2 | 2.6±0.7 | 14.0±2.7 | 6.1±1.1^ab^ | 23.9±4.5^b^ |

Data are given as group means ± SEM, n=12 (6 pens per diet with samples from 2 broilers per pen per treatment). Statistical analysis was performed by multiple T-tests with correction for multiple comparisons using the Holm-Šidák method. Differences in superscripts within columns indicate differences, at p < 0.05, between groups.

Table S5. Inositol phosphate levels (nmol/g wwt) in ileum segments of day 21 broilers

| Diet | InsP_3_ | InsP_4_ | InsP_5_ | InsP_6_ | ∑InsP |
| --- | --- | --- | --- | --- | --- |
| Control | 1.0±0.1 | 10.2±1.2^a^ | 28.1±2.5^a^ | 18.6±7.9 | 58.0±7.8^a^ |
| 2g/kg inositol | 0.4±0.2 | 2.2±5.6 ^b^ | 5.6±2.7^b^ | 3.3±0.8 | 11.5±2.7^b^ |
| Phy500 | 1.4±0.5 | 10.8±3.0^ab^ | 21.2±2.1^a^ | 11.5±2.4 | 45.0±6.6^ab^ |
| Phy6000 | 1.2±0.3 | 4.6±0.5 ^b^ | 12.6±1.4^b^ | 6.1±0.9 | 24.6±2.4^b^ |
| Control TiO_2_ | 0.8±0.1 | 5.4±0.9^ab^ | 17.0±1.8^b^ | 40.3±21.0 | 63.5±20.0^ab^ |
| 2g/kg inositol TiO_2_ | 1.8±0.2 | 7.9±1.3^ab^ | 19.1±2.7^b^ | 18.3±6.7 | 47.1±7.3^ab^ |
| Phy500 TiO_2_ | 0.6±0.1 | 5.6±0.4^ab^ | 17.3±1.7^b^ | 21.4±8.5 | 44.9±8.9^ab^ |
| Phy6000 TiO_2_ | 1.4±0.3 | 8.3±1.4^ab^ | 18.4±2.7^b^ | 5.9±0.6 | 34.0±4.2^ab^ |

Data are given as group means ± SEM, n=12 (6 pens per diet with samples from 2 broilers per pen per treatment). Statistical analysis was performed by multiple T-tests with correction for multiple comparisons using the Holm-Šidák method. Differences in superscripts within columns indicate differences, at p < 0.05, between groups.

Table S6: Dietary treatments and Test Substance inclusion rates, after ^4^

| Dietary Treatment | Test Substance inclusion rates to the basal diet | | |
| --- | --- | --- | --- |
|  | Phytase (g/tonne) | ^13^C Inositol mix (g/tonne) | TiO_2_ (g/tonne) |
| Control | - | - | - |
| 2 g/kg Ins | - | 2000 | - |
| Phy500 | 100 | - | - |
| Phy6000 | 1200 | - | - |
| Control TiO_2_ | - | - | 5000 |
| 2 g/kg Ins TiO_2_ | - | 2000 | 5000 |
| Phy500 TiO_2_ | 100 | - | 5000 |
| Phy6000 TiO_2_ | 1200 | - | 5000 |

References

1 Sommerfeld, V., Kunzel, S., Schollenberger, M., Kuhn, I., Rodehutscord, M. Influence of phytase or *myo*-inositol supplements on performance and phytate degradation products in the crop, ileum, and blood of broiler chickens. *Poult Sci* **97**, 920-929 (2018). 10.3382/ps/pex390

2 Sommerfeld, V., Schollenberger, M., Kuhn, I., Rodehutscord, M. Interactive effects of phosphorus, calcium, and phytase supplements on products of phytate degradation in the digestive tract of broiler chickens. *Poult Sci* **97**, 1177-1188 (2018). 10.3382/ps/pex404

3 Greiner, R., Konietzny, U., Jany, K.D. Purification and characterization of two phytases from *Escherichia coli*. *Arch Biochem Biophys* **303**, 107-113 (1993). 10.1006/abbi.1993.1261

4 Sprigg, C., Leftwich, P.T., Burton, E., Scholey, D., Bedford, M.R., Brearley, C.A. Accentuating the positive and eliminating the negative: Efficacy of TiO_2_ as digestibility index marker for poultry nutrition studies. *PLoS One* **18**, e0284724 (2023). 10.1371/journal.pone.0284724
