# Supplementary material for: Phosphorus-specific, liquid chromatography inductively coupled plasma mass-spectrometry for analysis of inositol phosphate and inositol pyrophosphate metabolism
Source: Biochem J. Author manuscript; Available in PMC 2025 Oct 12. (PMC7618242; doi:10.1042/BCJ20253151)
Supplement: Supplementary file 1 [file EMS209519-supplement-Supplementary_Infirmation.pdf]

Supplementary Information

Phosphorus-specific, liquid chromatography inductively coupled plasma mass-spectrometry for analysis of inositol phosphate and inositol pyrophosphate metabolism

[1]Colleen Sprigg, [1]Hayley Whitfield, [1]Philip T. Leftwich, [2]Hui-Fen Kuo, [2]Tzyy-Jen Chiou, [3]Adolfo Saiardi, [4]Megan L. Shipton, [4]Andrew M. Riley, [4]Barry V.L. Potter, [5]Dawn Scholey, [5]Emily Burton, [6]Mike R. Bedford, [1]Charles A. Brearley

[1]School of Biological Sciences, University of East Anglia, Norwich Research Park, Norwich NR4 7TJ, UK;

[2]Agricultural Biotechnology Research Center, Academia Sinica, Taipei 115, Taiwan;

[3]Laboratory for Molecular Cell Biology, University College London, London WC1E 6BT, UK;

[4]Medicinal Chemistry & Drug Discovery, Department of Pharmacology, University of Oxford, Mansfield Road, Oxford OX1 3QT, UK;

[5]School of Animal, Rural and Environmental Sciences, Nottingham Trent University, Southwell, Nottingham NG25 0QF, UK;

[6]AB Vista, Marlborough SN8 4AN, UK

Michael R. Bedford orcid.org/0000-0002-5308-4290

Charles A. Brearley; orcid.org/0000- 0001-6179-9109

Emily Burton; orcid.org/0000-0003-2784-6922

Tzyy-Jen Chiou; orcid.org/0000-0001-5953-4144

Philip T. Leftwich; orcid.org/0000-0001-9500-6592

Barry V.L. Potter; orcid.org/0000-0003-3255-9135

Andrew M. Riley; orcid.org/0000-0001-9003-3540

Adolfo Saiardi; orcid.org/0000-0002-4351-0081

Megan L. Shipton; orcid.org/0000-0002-9982-0927

Colleen Sprigg; orcid.org/0000-0001-7755-4245

Hayley L. Whitfield; orcid.org/0000-0001-6003-7874

Figure S1. LC-ICP-MS analysis of $InsP_6$ digestion in the avian gastrointestinal tract.

Figure S2. Biological variability and reproducibility of LC-ICP-MS

Table S1. Statistical analysis of phytase inclusion in diet on inositol phosphate content of gut tissues

Table S2. Analysis of effect of phytase inclusion in diet on $InsP_5$: $InsP_6$ ratio of gut tissues

Table S3. Inositol phosphate levels in duodenal segments of chicken

Table S4. Inositol phosphate levels in jejunum segments of chicken

Table S5. Inositol phosphate levels in ileum segments of chicken

Table S6. Dietary treatments and Test Substance inclusion rates

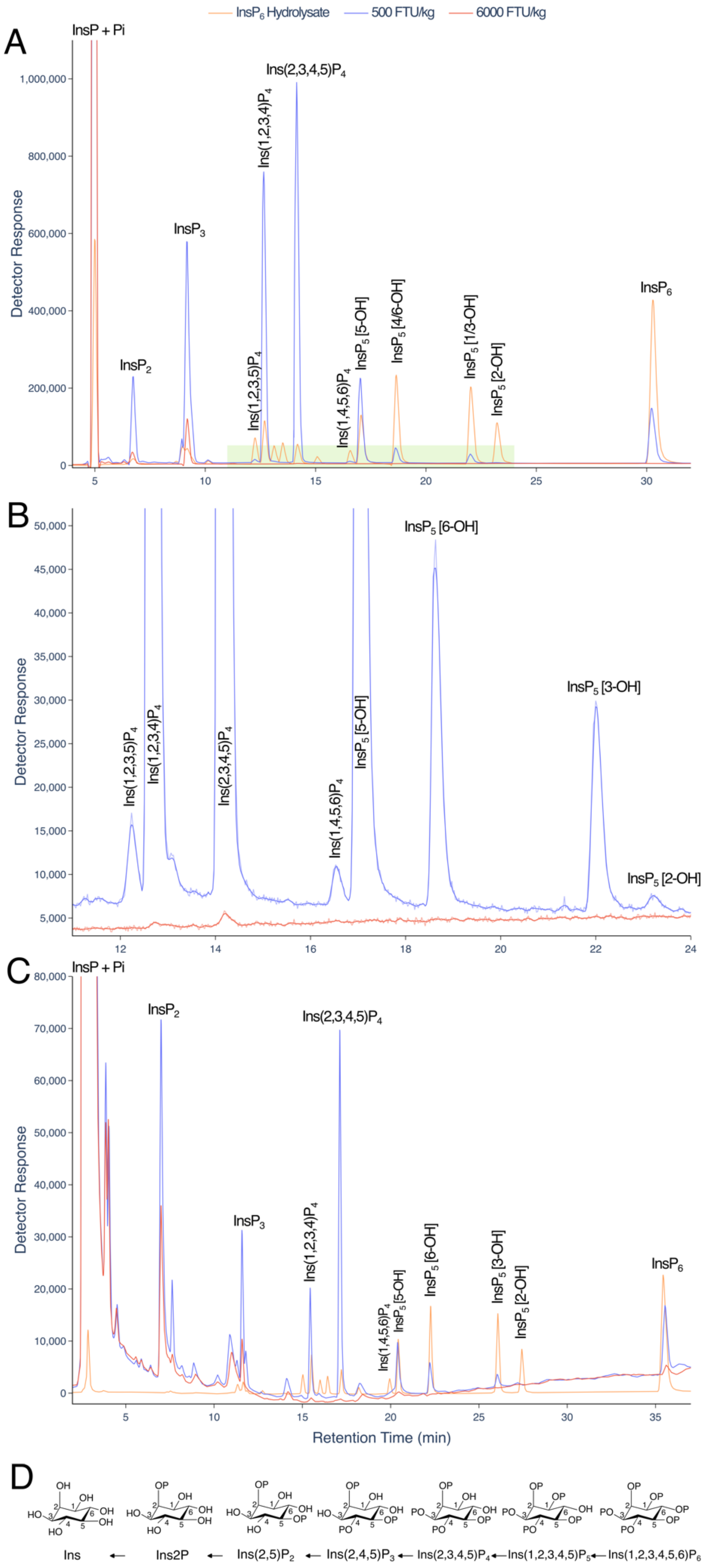
InsP6 Hydrolysate
500 FTU/kg
6000 FTU/kg
A
B
C
D
Detector Response
Retention Time (min)
InsP + Pi
InsP2
InsP3
Ins(1,2,3,5)P4
Ins(1,2,3,4)P4
Ins(2,3,4,5)P4
Ins(1,4,5,6)P4
InsP5 [5-OH]
InsP5 [4/6-OH]
InsP5 [1/3-OH]
InsP5 [2-OH]
InsP5 [6-OH]
InsP5 [3-OH]
InsP6
Ins ← Ins2P ← Ins(2,5)P2 ← Ins(2,4,5)P3 ← Ins(2,3,4,5)P4 ← Ins(1,2,3,4,5)P5 ← Ins(1,2,3,4,5,6)P6

39

Figure S1. LC-ICP-MS analysis of $InsP_6$ digestion in the avian gastrointestinal tract. **A**. LC-ICP-MS of lumenal gizzard content of birds fed a diet containing low (500 FTU/kg) (blue) or high (6000 FTU/kg) (red) phytase, beside a hydrolysate of $InsP_6$ (orange). **B**. An expansion of the $InsP_4$ and $InsP_5$ region, indicated by the pale green panel, of the 500 FTU/kg and 6000 FTU/kg traces shown A. **C.** LC-UV analysis of lumenal content of gizzard of birds fed a diet containing low (500 FTU/kg) (blue) or high (6000 FTU/kg) (red) phytase, beside a hydrolysate of $InsP_6$ (orange). **D**. Pathway of degradation of $InsP_6$ by 6-phytase after [1,2,3]. Final digestive dephosphorylation of InsP is likely catalysed by alkaline phosphatase of the mucosal epithelia. For all panels, samples were resolved on a CarboPac PA200 column eluted with methanesulfonic acid. Separations of gut lumen inositol phosphates matching the resolution shown (A, B, C) have been observed on more than 1000 occasions at varying phytase dose on CarboPac PA200 coupled to LC-UV. For the ICP analysis shown, various digesta samples have been analysed on more than 10 occasions with equivalent resolution and detector response.

A

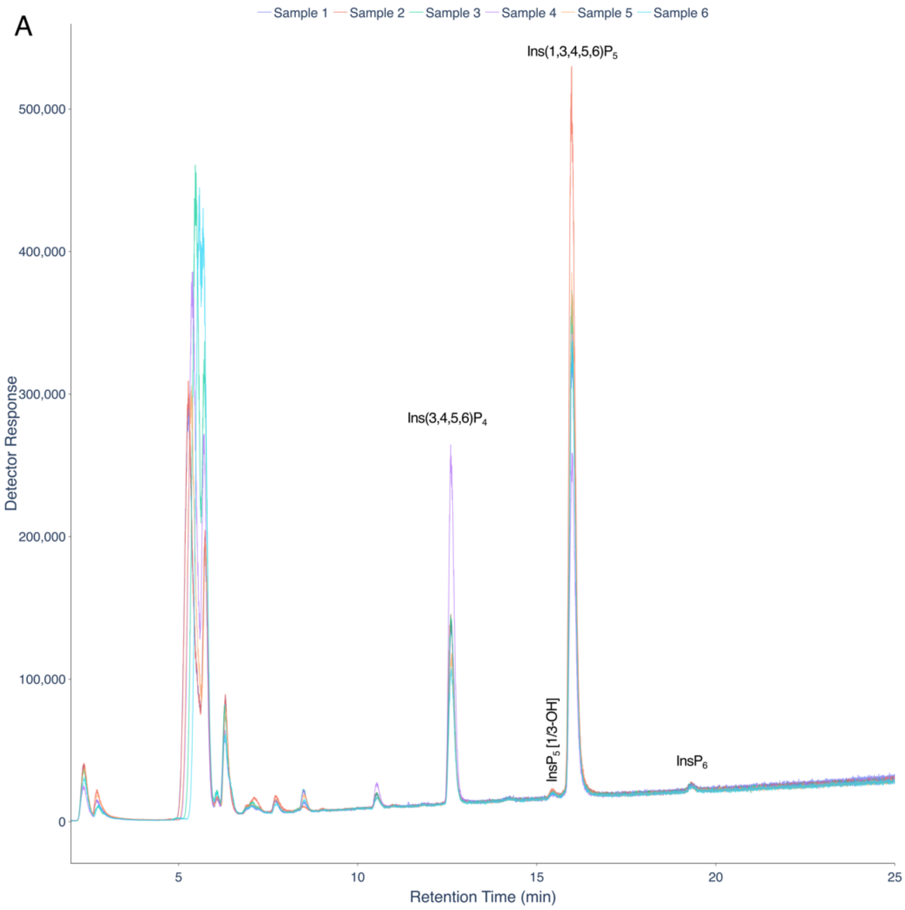

Sample 1
Sample 2
Sample 3
Sample 4
Sample 5
Sample 6
Ins(1,3,4,5,6)P5
Ins(3,4,5,6)P4
InsP5 [1/3-OH]
InsP6
Detector Response
Retention Time (min)

B

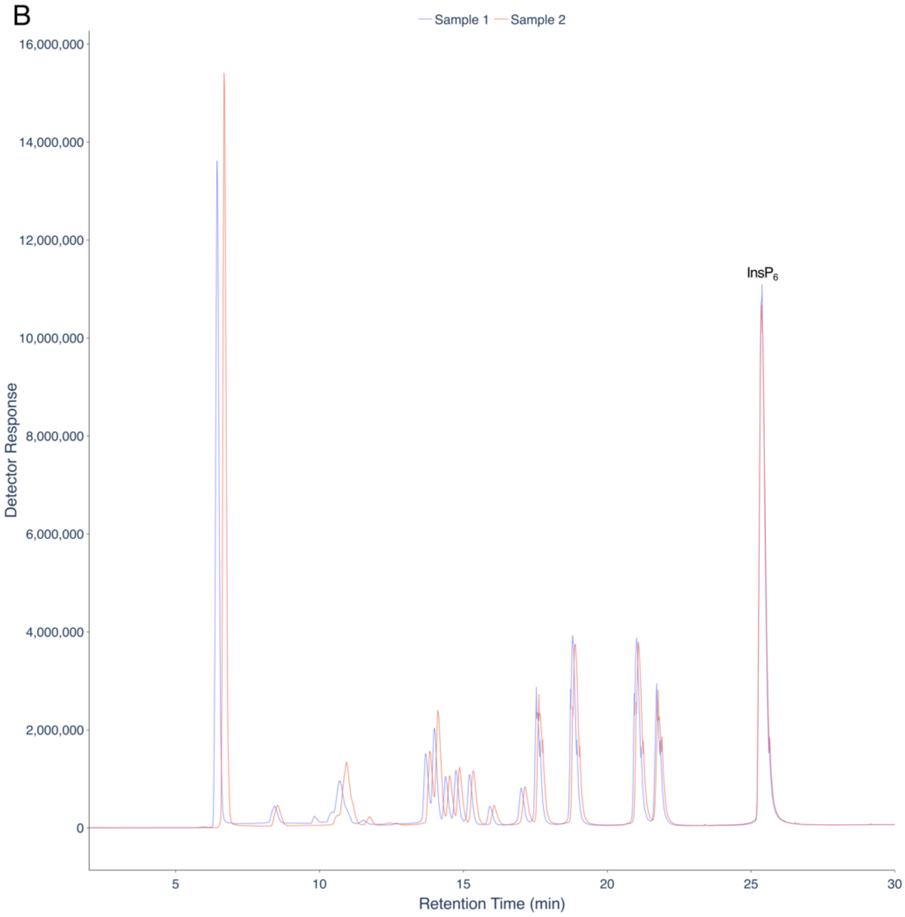

Sample 1
Sample 2
InsP6
Detector Response
Retention Time (min)

Figure S2. **Biological variability and reproducibility of LC-ICP-MS**. Inositol phosphate extracts were prepared from blood samples obtained from six 35 d old chickens raised in different pens and fed a control diet. Whole blood was extracted in $HClO_4$, diluted with NaF-EDTA and analysed by LC-ICP-MS. The samples were analysed at the end of a set of 25 consecutive injections. For Ins(3,4,5,6)P_4 and Ins(1,3,4,5,6)P_5, with mean retention time (and coefficient of variation) 12.06 min (0.070) and 15.987 min (0.011), the two peaks had areas with mean (and standard error) of 1,516,234 (244,855) and 4,191,724 (425,478), respectively. B) Two replicate injections of an InsP_6 hydrolysate sample were analysed at the end of a set of more than 50 consecutive injections. For these, the InsP_6 peak area (counts.min) were 161,430,343 and 171,113,2621. The InsP_5 peaks (not labelled), similarly, differed by less than 4% of the mean value for the duplicate measurements. In a separate experiment, three replicate injections of a different InsP_6 hydrolysate sample gave peak areas (counts.min) for InsP_6 of mean 2,041,016 and standard error 23,460. Different HCl gradients were used for A and B.

Table S1. Analysis of effect of phytase inclusion in diet on inositol phosphate content of duodenum, jejunum and ileum tissue, analysed by a linear mixed-effects model with a log transformation (+1) applied to InsP measures. Phytase was added, or not, at 500 or 6000 FTU/kg. Inositol was added at 2g/kg.

	**log(value + 1)**		
Predictors	*Estimates*	*CI*	*p*
(Intercept)	0.43	0.24 – 0.62	**<0.001**
Control [Inositol_added]	0.42	0.18 – 0.66	**0.001**
Control [Phytase 500]	0.21	-0.03 – 0.45	0.092
Control [Phytase 6000]	0.29	0.05 – 0.53	**0.019**
$InsP_4$	1.02	0.82 – 1.23	**<0.001**
$InsP_5$	2.51	2.30 – 2.71	**<0.001**
$InsP_6$	2.31	2.11 – 2.52	**<0.001**
Tissue [Jejunum]	0.25	0.06 – 0.44	**0.011**
Tissue [Ileum]	0.16	-0.03 – 0.35	0.098
Titanium [Yes]	0.11	0.00 – 0.22	**0.041**
Control [Inositol added] × $InsP_4$	-0.47	-0.71 – -0.23	**<0.001**
Control [Phytase 500] × $InsP_4$	0.05	-0.19 – 0.29	0.687
Control [Phytase 6000] × $InsP_4$	-0.21	-0.45 – 0.03	0.081
Control [Inositol added] × $InsP_5$	-0.57	-0.81 – -0.34	**<0.001**
Control [Phytase 500] × $InsP_5$	0.01	-0.22 – 0.25	0.918
Control [Phytase 6000] × $InsP_5$	-0.42	-0.65 – -0.18	**0.001**
Control [Inositol added] × $InsP_6$	-0.71	-0.95 – -0.47	**<0.001**
Control [Phytase 500] × $InsP_6$	-0.33	-0.56 – -0.09	**0.007**
Control [Phytase 6000] × $InsP_6$	-1.10	-1.34 – -0.86	**<0.001**
$InsP_4$ × Tissue [Jejunum]	-0.23	-0.43 – -0.02	**0.031**
$InsP_5$ × Tissue [Jejunum]	-0.20	-0.41 – 0.00	0.054
$InsP_6$ × Tissue [Jejunum]	-0.01	-0.22 – 0.19	0.899
$InsP_4$ × Tissue [Ileum]	0.37	0.16 – 0.58	**<0.001**
$InsP_5$ × Tissue [Ileum]	-0.14	-0.35 – 0.06	0.166
$InsP_6$ × Tissue [Ileum]	-0.17	-0.37 – 0.04	0.113
Control [Inositol added] × Tissue [Jejunum]	-0.20	-0.41 – 0.00	0.054
Control [Phytase 500] × Tissue [Jejunum]	-0.36	-0.57 – -0.16	**0.001**
Control [Phytase 6000] × Tissue [Jejunum]	-0.32	-0.52 – -0.11	**0.003**
Control [Inositol added] × Tissue [Ileum]	-0.54	-0.74 – -0.33	**<0.001**
Control [Phytase 500] × Tissue [Ileum]	-0.25	-0.45 – -0.04	**0.018**
Control [Phytase 6000] × Tissue [Ileum]	-0.18	-0.39 – 0.03	0.086
**Random Effects**			
σ^2	0.26		
$\tau_{00\ id}$	0.05		
ICC	0.16		
N_{id}	96		

Observations	1152
Marginal R^2 / Conditional R^2	0.705 / 0.753

Table S2. Analysis of effect of phytase inclusion in diet on $InsP_5$: $InsP_6$ ratio of duodenum, jejunum and ileum tissue, analysed by a linear mixed-effects model with a square root transformation applied to ratios. Phytase was added, or not, at 500 or 6000 FTU/kg. Inositol was added at 2g/kg.

	sqrt($InsP_5$: $InsP_6$)		
Predictors	*Estimates*	*CI*	*p*
(Intercept)	1.28	1.10 – 1.47	**<0.001**
Control [Inositol added]	-0.01	-0.26 – 0.23	0.918
Control [Phytase 500]	0.17	-0.07 – 0.42	0.169
Control [Phytase 6000]	0.40	0.15 – 0.65	**0.002**
Tissue [Jejunum]	-0.21	-0.45 – 0.02	0.076
Tissue [Ileum]	0.12	-0.12 – 0.35	0.335
Titanium [Yes]	-0.10	-0.22 – 0.01	0.062
Control [Inositol added] × Tissue [Jejunum]	0.24	-0.10 – 0.57	0.164
Control [Phytase 500] × Tissue [Jejunum]	0.14	-0.19 – 0.48	0.395
Control [Phytase 6000] × Tissue [Jejunum]	0.20	-0.13 – 0.53	0.240
Control [Inositol added] × Tissue [Ileum]	0.02	-0.31 – 0.36	0.897
Control [Phytase 500] × Tissue [Ileum]	-0.10	-0.44 – 0.23	0.541
Control [Phytase 6000] × Tissue [Ileum]	-0.10	-0.44 – 0.23	0.536
**Random Effects**			
σ^2	0.17		
$\tau_{00\ id}$	0.02		
$\tau_{00\ Diet}$	0.00		
N_{id}	96		
N_{Diet}	8		
Observations	286		
Marginal R^2 / Conditional R^2	0.173 / NA		

Table S3. Inositol phosphate levels (nmol/g wwt) in duodenal segments of day 21 broilers

Diet	$InsP_3$	$InsP_4$	$InsP_5$	$InsP_6$	∑InsP
Control	0.6±0.1^{b}	4.1±0.7	21.2±4.2	14.4±3.9	40.3±8.4
2g/kg inositol	1.1±0.2^{ab}	2.6±0.5	12.0±1.2	13.5±4.7	29.2±5.2
Phy500	0.8±0.1^{ab}	5.0±0.6	26.1±2.7	13.0±1.0	44.9±3.3
Phy6000	1.2±0.1^{ab}	5.8±0.5	26.7±2.2	9.2±0.9	42.9±3.1
Control TiO_2	0.9±0.2^{ab}	4.6±0.5	24.1±2.6	22.7±4.2	52.2±3.6
2g/kg inositol TiO_2	2.8±0.2^{aa}	4.0±0.5	21.7±2.3	21.1±6.7	49.7±8.1
Phy500 TiO_2	1.1±0.2^{ab}	5.3±0.4	27.9±1.8	16.5±2.6	50.7±4.2
Phy6000 TiO_2	1.1±0.2^{ab}	3.0±0.6	12.4±1.6	13.5±0.5	21.8±2.1

Data are given as group means ± SEM, n=12 (6 pens per diet with samples from 2 broilers per pen per treatment). Statistical analysis was performed by multiple T-tests with correction for multiple comparisons using the Holm-Šidák method. Differences in superscripts within columns indicate differences, at $p < 0.05$, between groups.

Table S4. Inositol phosphate levels (nmol/g wwt) in jejunum segments of day 21 broilers

Diet	$InsP_3$	$InsP_4$	$InsP_5$	$InsP_6$	∑InsP
Control	1.2±0.1	3.8±0.6	19.5±2.3	33.5±8.9^{a}	58.0±9.8^{a}
2g/kg inositol	0.8±0.2	2.2±0.5	12.6±1.6	20.9±8.0^{ab}	36.4±8.2^{ab}
Phy500	1.4±0.3	4.1±0.4	22.3±3.2	11.4±2.5^{b}	39.2±4.6^{ab}
Phy6000	1.8±0.6	5.0 ±1.3	15.7±2.1	5.8±0.8^{b}	28.3±2.8^{b}
Control TiO_2	1.2±0.2	3.8±0.6	22.6±3.1	29.7±6.0^{ab}	57.3±6.7^{ab}
2g/kg inositol TiO_2	1.7±0.2	4.5±0.3	20.2±1.4	14.8±3.2^{ab}	41.2±3.8^{ab}
Phy500 TiO_2	0.9±0.2	2.7±0.5	16.1±1.9	16.2±3.0^{ab}	25.9±4.2^{ab}
Phy6000 TiO_2	1.0±0.2	2.6±0.7	14.0±2.7	6.1±1.1^{ab}	23.9±4.5^{b}

Data are given as group means ± SEM, n=12 (6 pens per diet with samples from 2 broilers per pen per treatment). Statistical analysis was performed by multiple T-tests with correction for multiple comparisons using the Holm-Šidák method. Differences in superscripts within columns indicate differences, at $p < 0.05$, between groups.

Table S5. Inositol phosphate levels (nmol/g wwt) in ileum segments of day 21 broilers

Diet	$InsP_3$	$InsP_4$	$InsP_5$	$InsP_6$	∑InsP
Control	1.0±0.1	10.2±1.2^{a}	28.1±2.5^{a}	18.6±7.9	58.0±7.8^{a}
2g/kg inositol	0.4±0.2	2.2±5.6 ^{b}	5.6±2.7^{b}	3.3±0.8	11.5±2.7^{b}
Phy500	1.4±0.5	10.8±3.0^{ab}	21.2±2.1^{a}	11.5±2.4	45.0±6.6^{ab}
Phy6000	1.2±0.3	4.6±0.5 ^{b}	12.6±1.4^{b}	6.1±0.9	24.6±2.4^{b}
Control TiO_2	0.8±0.1	5.4±0.9^{ab}	17.0±1.8^{b}	40.3±21.0	63.5±20.0^{ab}
2g/kg inositol TiO_2	1.8±0.2	7.9±1.3^{ab}	19.1±2.7^{b}	18.3±6.7	47.1±7.3^{ab}
Phy500 TiO_2	0.6±0.1	5.6±0.4^{ab}	17.3±1.7^{b}	21.4±8.5	44.9±8.9^{ab}
Phy6000 TiO_2	1.4±0.3	8.3±1.4^{ab}	18.4±2.7^{b}	5.9±0.6	34.0±4.2^{ab}

Data are given as group means ± SEM, n=12 (6 pens per diet with samples from 2 broilers per pen per treatment). Statistical analysis was performed by multiple T-tests with correction for multiple comparisons using the Holm-Šidák method. Differences in superscripts within columns indicate differences, at $p < 0.05$, between groups.

Table S6: Dietary treatments and Test Substance inclusion rates, after [4]

Dietary Treatment	Test Substance inclusion rates to the basal diet		
	Phytase (g/tonne)	^{13}C Inositol mix (g/tonne)	TiO_2 (g/tonne)
Control	-	-	-
2 g/kg Ins	-	2000	-
Phy500	100	-	-
Phy6000	1200	-	-
Control TiO_2	-	-	5000
2 g/kg Ins TiO_2	-	2000	5000
Phy500 TiO_2	100	-	5000
Phy6000 TiO_2	1200	-	5000

References

1 Sommerfeld, V., Kunzel, S., Schollenberger, M., Kuhn, I., Rodehutscord, M. Influence of phytase or *myo*-inositol supplements on performance and phytate degradation products in the crop, ileum, and blood of broiler chickens. *Poult Sci* **97**, 920-929 (2018). 10.3382/ps/pex390

2 Sommerfeld, V., Schollenberger, M., Kuhn, I., Rodehutscord, M. Interactive effects of phosphorus, calcium, and phytase supplements on products of phytate degradation in the digestive tract of broiler chickens. *Poult Sci* **97**, 1177-1188 (2018). 10.3382/ps/pex404

3 Greiner, R., Konietzny, U., Jany, K.D. Purification and characterization of two phytases from *Escherichia coli*. *Arch Biochem Biophys* **303**, 107-113 (1993). 10.1006/abbi.1993.1261

4 Sprigg, C., Leftwich, P.T., Burton, E., Scholey, D., Bedford, M.R., Brearley, C.A. Accentuating the positive and eliminating the negative: Efficacy of TiO_2 as digestibility index marker for poultry nutrition studies. *PLoS One* **18**, e0284724 (2023). 10.1371/journal.pone.0284724
